# Synthesis, Structure, and Electric Conductivity of Higher Hydrides of Ytterbium at High Pressure

**DOI:** 10.1021/acs.inorgchem.2c00405

**Published:** 2022-06-01

**Authors:** Tomasz Jaroń, Jianjun Ying, Marek Tkacz, Adam Grzelak, Vitali B. Prakapenka, Viktor V. Struzhkin, Wojciech Grochala

**Affiliations:** † Centre of New Technologies, University of Warsaw, Banacha 2c, 02-097 Warsaw, Poland; ‡ Geophysical Laboratory, Carnegie Institution of Washington, 5251 Broad Branch Road NW, Washington, District of Columbia 20015, United States; § Faculty of Chemistry, University of Warsaw, Pasteura 1, 02-089 Warsaw, Poland; ∥ HPCAT, Geophysical Laboratory, Carnegie Institution of Washington, Argonne, Illinois 60439, United States; ⊥ Institute for Physical Chemistry, Polish Academy of Science, 01-224 Warsaw, Poland; # Consortium for Advanced Radiation Sources, The University of Chicago, Chicago, Illinois 60637, United States; ∇ Center for High Pressure Science and Technology Advanced Research, Shanghai 201203, China

## Abstract

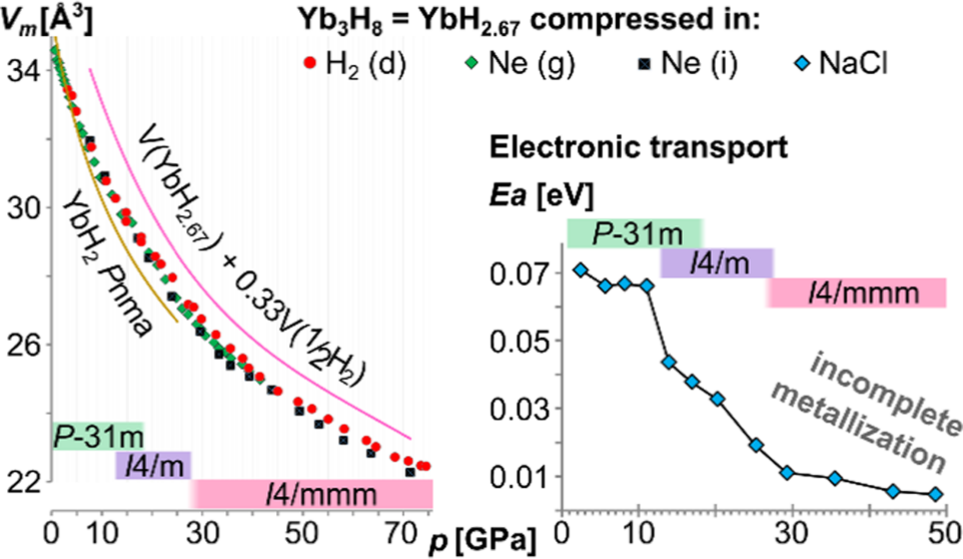

While most of the rare-earth metals readily form trihydrides, due to increased stability of the filled 4f electronic shell for Yb(II), only YbH_2.67_, formally corresponding to Yb^II^(Yb^III^H_4_)_2_ (or Yb_3_H_8_), remains the highest hydride of ytterbium. Utilizing the diamond anvil cell methodology and synchrotron powder X-ray diffraction, we have attempted to push this limit further *via* hydrogenation of metallic Yb and Yb_3_H_8_. Compression of the latter has also been investigated in a neutral pressure-transmitting medium (PTM). While the *in situ* heating of Yb facilitates the formation of YbH_2+*x*_ hydrides, we have not observed clear qualitative differences between the systems compressed in H_2_ and He or Ne PTM. In all of these cases, a sequence of phase transitions occurred within *ca.* 13–18 GPa (*P*3̅1*m*–*I*4/*m* phase) and around 27 GPa (to the *I*4/*mmm* phase). The molecular volume of the systems compressed in H_2_ PTM is *ca.* 1.5% larger than of those compressed in inert gases, suggesting a small hydrogen uptake. Nevertheless, hydrogenation toward YbH_3_ is incomplete, and polyhydrides do not form up to the highest pressure studied here (*ca.* 75 GPa). As pointed out by electronic transport measurements, the mixed-valence Yb_3_H_8_ retains its semiconducting character up to >50 GPa, although the very low remnant activation energy of conduction (<5 meV) suggests that metallization under further compression should be achievable. Finally, we provide a theoretical description of a hypothetical stoichiometric YbH_3_.

## Abstract 2

Hydrogenation of Yb and Yb_3_H_8_ has been attempted under high pressure (≤75 GPa); the latter compound has also been investigated in Ne and He. The same sequence of phase transitions observed in all of these systems, with only minor differences in molar volume (1.5%), indicates that the limiting composition remains not far from YbH_2.67_. The latter retains its semiconducting character up to >50 GPa, with a very low remnant activation energy of conduction (<5 meV).

## Introduction

The enormous interest in diverse hydrogen-rich systems manifested in the last few decades can be linked mainly to two topics: energy storage in chemical compounds and phonon-driven superconductivity (SC), depending on the nature of systems studied. Due to the high gravimetric efficiency required for energy storage in most potential applications, the first area has been dominated by materials composed predominantly of light elements such as Li, B, Al, etc.^1−4^ On the other hand, the hydrides that have been studied as potential superconductors are not limited in this respect and their components cover virtually the entire periodic table.^5−8^

The recent upsurge in the search for high-temperature SC in hydrides under high pressure has been stimulated by Neil Ashcroft’s idea of “chemical precompression” of hydrogen contained in hydrogen-rich chemical compounds.^9^ In such hydrogen-dominated systems (in terms of an atomic fraction), the hydrogen sublattice is expected to contribute to the metallic state at drastically lower pressures compared to elemental hydrogen. This would finally allow for taking an advantage of the large phonon frequencies related to the lightweight hydrogen atoms to raise the critical temperature of SC. SC might occur provided the system features a high density of electronic states at the Fermi level and strong electron–phonon coupling. As a consequence of this concept, many binary and even more complex hydride-based systems have been screened theoretically,^10^ leading ultimately to experimental discovery of superconductivity in some of them. Several hydrides, especially those containing the p-block and rare-earth (RE) elements,^7^ were indicated as particularly promising by theoretical predictions, *e.g.*, H_3_S^11,12^ or YH_10_ and LaH_10_.^13,14^ The experimental verification of these predictions led to reports of strikingly high critical temperatures of superconductivity (*T*_C_): compressed H_2_S forming a superconducting H_3_S phase of *T*_C_ = 203 K at 155 GPa,^15^ LaH_10_ of *T*_C_ = 250–260 K upon compression to *ca.* 200 GPa,^16−18^ an incompletely identified C–S–H material synthesized in a diamond anvil cell (DAC) of *T*_C_ = 288 K at 267 GPa,^19^ or YH_6_ of *T*_C_ = 220 K at 166–183 GPa^20,21^ and YH_9_ of *T*_C_ = 243–262 K at 182–201 GPa.^21,22^

Considering the RE-based systems, in these superhydrides, the hydrogen sublattice is usually composed of clathrate-like and other complex geometrical motifs or contains mixed molecular and atomic units.^5,10,23^ While the polyhydrides of exotic compositions may form in a megabar pressure range, they are preceded by more usual stoichiometries appearing at much lower pressures. Some of the latter also superconduct, as has been verified experimentally or as expected based on computational screening.^7^ Obviously, lowering the pressure of synthesis of a superconducting hydride or the metastability of such phases under as close to ambient conditions as possible would be beneficial for more thorough macroscopic studies and possible applications. Recently, such high-temperature superconducting phases have been found for the hydrides based on lanthanides below the megabar pressure region.^24,25^

With the exception of Eu and Yb, all lanthanides are easily hydrogenated at room temperature, absorbing up to 300 mol % hydrogen to form trihydrides, LnH_3_, already under the pressure of a few atmospheres;^26^ see also Figure S1 in the Supporting Information (SI). However, due to the stabilization of half-filled or filled 4f electronic shells for Eu(II) and Yb(II), respectively, oxidation of these elements to the trivalent state by hydrogen is much more difficult. While the corresponding dihydrides can be readily prepared,^26^ further absorption of hydrogen requires significantly higher pressure. The uptake of hydrogen by EuH_2_ occurs only above 8.7 GPa, as has been reported by Matsuoka et al. based on X-ray diffraction and Eu–Mössbauer spectroscopy measurements.^27^ At this pressure range, a tetragonal EuH_2+*x*_ containing trivalent Eu^3+^ starts to form, and the latter remains the only detected oxidation state of Eu above 12.5 GPa.^28^ Very recently, Semenok et al.^29^ reported that EuH_9_ is present in the samples already at 74 GPa, while the other europium superhydrides were detected within the range 74–130 GPa, some of them showing antiferromagnetic ordering. Unfortunately, the lower-pressure regime has not been studied in this work, leaving the gap between *ca.* 50 GPa for which EuH_2+*x*_ phases were reported and 74 GPa with the superhydride phases already formed.

**Figure 1:**
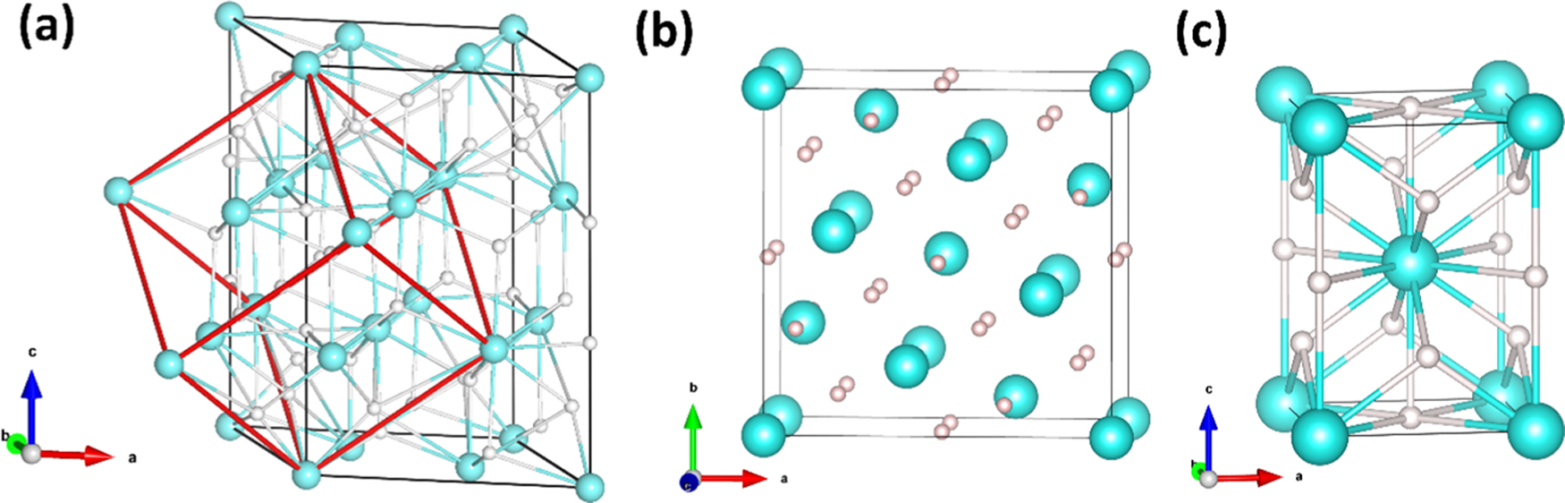
Overview of the crystal structures of LnH_2+*x*_, Ln = Yb, Eu: (a) Yb_3_H_8_ (YbH_2.67_) *P*3̅1*m*;^33^ (b) EuH_2+*x*_*I*4/*m*; and (c) EuH_2+*x*_*I*4/*mmm*.^27^

In the case of ytterbium, some early reports indicated preparation of cubic YbH_2+*x*_, *x* < 0.7 after reaction of this metal with hydrogen pressurized to *ca.* 120 bar.^30−32^ These findings have been clarified by Auffermann, who prepared the metastable higher hydride of ytterbium *via* a high-pressure, high-temperature reaction (200–3200 bar, >600 K).^33^ While the major features of the powder X-ray diffraction pattern of YbH_2+*x*_ are compatible with a face-centered cubic (fcc) unit cell reported earlier, application of powder neutron diffraction allowed for proper identification and refinement of its crystal structure. It appeared that the prepared compound can be described as YbH_2.67_, *i.e.*, Yb_3_H_8_ or Yb^II^(Yb^III^H_4_)_2_, and it crystallizes in a trigonal unit cell with close packing of the nine ytterbium atoms (stacking sequence: ...ABC...), while hydrogen occupies all of the tetrahedral and 2/3 of the octahedral holes, and no disorder is observed in the structure. The crystal structures of the higher hydrides of Yb and Eu relevant to the current study are summarized in Figure 1.

The case of Yb(III) is special.^2^ The vicinity of 4f shell closure for the 4f^13^ electron configuration of Yb^III^ resembles the 3d^9^ configuration of parent oxocuprate superconductors, and the early DFT calculations indicated the possibility of the significant contribution of H 1s states to the electron density at the Fermi level even for the stoichiometric Yb(III) hydrides.^34^ Markedly, the only hole in the f electron set usually resides on the orbital, which forms sigma* states with H(1s), which is analogous to a hole in the d set of Cu(II) residing in sigma* states with O(2p) in cuprates. Moreover, Yb(III) features a large magnetic moment of *ca.* 4.3–4.9 *m*_B_ corresponding formally to spin-1/2, which again renders it similar to Cu(II) in parent compounds of oxocuprate superconductors; thus, one might expect that electron doping could affect magnetism and lead to SC, just like for Cu(II) oxides. Last but not the least, more recent theoretical considerations suggest that besides yttrium and the lightweight lanthanides (Ln),^13,14,35^ the late lanthanides (Yb and Lu) should also form the superhydrides of *T*_C_ > 100 K.^36^

In the present work, we expand the chemistry of the Yb–H system far beyond the previously studied range of *ca.* 0.3 GPa. Using the DAC methodology, we investigate the reaction of metallic Yb with H_2_ and attempt further hydrogenation of Yb_3_H_8_ (YbH_2.67_). We discuss the observed phase transitions as well as the possibility of formation of the higher hydrides of ytterbium, *i.e.*, YbH_2+*x*_, especially with *x* > 0.67. We have also studied the electronic transport of the mixed-valence Yb_3_H_8_ in the function of external pressure.^37^

## Methods

### Experimental Section

Metallic Yb (99.9%, Sigma-Aldrich) and Au pressure standard (99.999%, Alfa Aesar) were loaded into the DAC. Diamonds of 200–300 μm culets and rhenium gaskets were used. Yb_3_H_8_ was prepared according to the literature procedure^33^ and analyzed at ambient conditions using powder X-ray diffraction, *cf.*Figure S2 and Table S1 in the SI. Ne, He, and H_2_, loaded at *ca.* 170 MPa in the custom-designed gas loading systems of GL CIW and GSE CARS APS,^38^ were applied as the pressure-transmitting medium (PTM).

High-pressure angle-dispersive X-ray diffraction (XRD) measurements were performed using the Advanced Photon Source synchrotron facility of the Argonne National Laboratory. The measurements were carried out on sectors 13ID-D and 16ID-B operating at wavelengths from 0.2952 to 0.4066 Å. The sample-to-detector distance and other geometrical parameters were calibrated using LaB_6_ or CeO_2_ standards. One of the samples of Yb in H_2_ PTM was heated by the double-sided laser systems available at the beamlines to the maximum temperatures of about 2100 K measured by fitting gray body thermal radiation.^39^ Heating was carried out in several pulses lasting for a few seconds; however, the overall amount of energy absorbed by the sample was not monitored. The heating was not uniform across the sample, which manifested as variable amounts of crystalline phases present in various areas of the sample.

The electronic transport properties were carried out in a custom, miniaturized diamond anvil cell in a classical four-electrode geometry, using a Quantum Design Physical Property Measurement System. NaCl was used as a PTM, and the pressure was measured using ruby fluorescence.

The two-dimensional diffraction images were analyzed and integrated using DIOPTAS software.^40^ X-cell was used for pattern indexing.^41^ The structures were refined in JANA2006.^42^ The pseudo-Voigt function with the Berar–Baldinozzi correction for asymmetry was utilized for modeling the diffraction peak shape. The background was corrected by Legendre polynomials. Further details of the crystal structure investigations may be obtained from the joint CCDC/FIZ Karlsruhe online deposition service: https://www.ccdc.cam.ac.uk/structures/ by quoting the deposition numbers CSD 2150168–2150171. The third-order Birch–Murnaghan equations of state (EoS) were fitted using the EoSFit7-GUI program.^43,44^

### Computational

Density functional theory (DFT) calculations were performed using CASTEP.^45^ Generalized gradient approximation (GGA) was used with the PBE functional adjusted for solids (PBEsol).^46^ As hydrides usually require a large cutoff, here the value of 700 eV was applied to lead to very good energy convergence. The density of the *k*-point grid was set at 0.04 Å^–1^; ultrasoft pseudopotentials generated on the fly were used as they provide more accurate lattice parameters. The YbH_3_ stoichiometry was assumed, and the *I*4/*mmm* structure type exhibited by YbH_2+*x*_ at elevated pressures was adopted. The sqrt2 × sqrt2 × 1 supercell (*Z* = 4) was applied to account for various magnetic ordering schemes; the formal spin of Yb(III) was used as the initial one in the first SCF step. For the magnetic structures, the DFT + *U* formalism was used, with *U* = 5 eV for the 4f electrons of Yb. Enthalpies of various magnetic models and that of a spin-unpolarized one were calculated at several pressure values. Electronic density of states (DOS) was computed for the lowest enthalpy solutions.

## Results and Discussion

**Figure 2:**
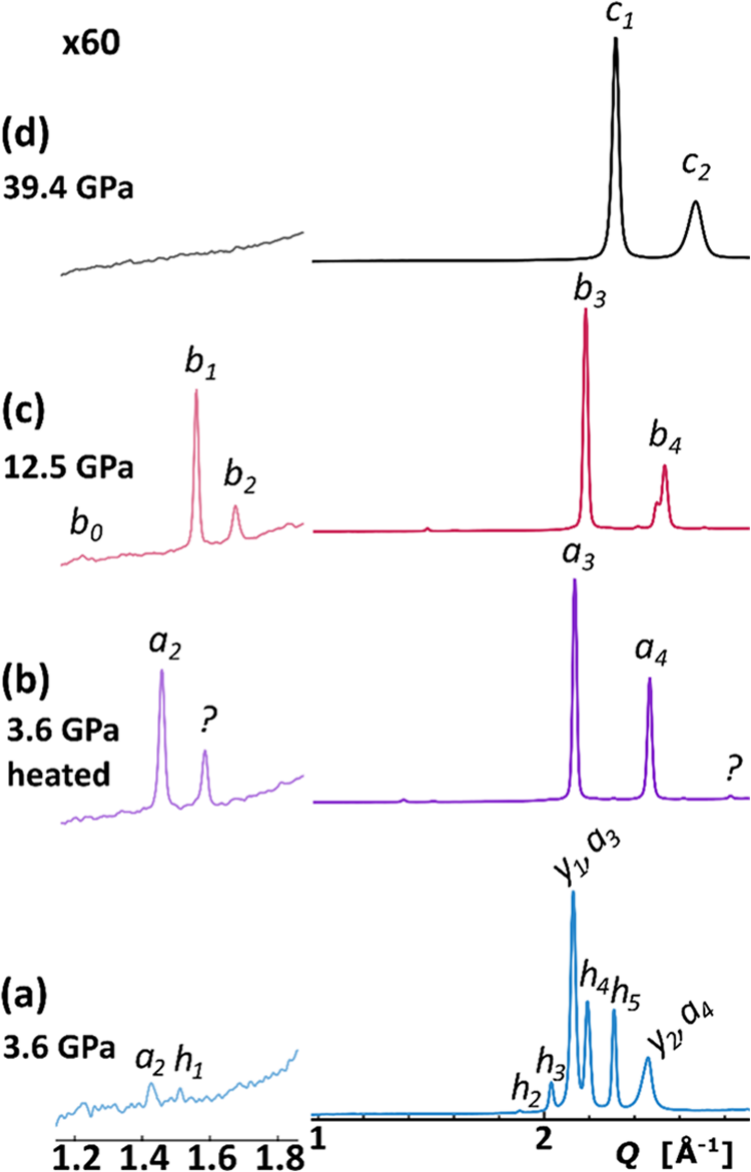
Diffraction patterns for the sample of Yb compressed in H_2_: (a) before and (b) after the laser heating at *ca.* 3.6 GPa; (c) after several rounds of laser heating (*ca.* 12.5 GPa); and (d) at *ca.* 39.4 GPa. The low-*Q* part has been additionally presented in 60× magnification of intensity. λ = 0.3344 Å. a-YbH_2+*x*_*P*3̅1*m*: 2-(1 0 1), 3-(0 0 3), and (2 1̅ 1), 4-(1 1 2); b-YbH_2+*x*_*I*4/*m*: 0-(1 1 0), 1-(1 0 1), 2-(2 0 0), 3-(2 1 1), 4-(0 0 2), and (3 1 0); c-YbH_2+*x*_*I*4/*mmm*: 1-(1 0 1), 2-(1 1 0), and (0 0 2); h-YbH_2_*Pnma*: 1-(1 0 1), 2-(0 0 2), 3-(0 1 1), 4-(1 0 2), and (2 0 0), 5-(1 1 1); y-Yb *Fm*3̅*m*: 1-(1 1 1), 2-(0 0 2).

As could be expected, the reaction between metallic ytterbium and hydrogen proceeds already at room temperature immediately after hydrogen loading to the cell. However, the unreacted metal is present in the whole pressure range studied, contributing to the strongest diffraction peaks (*cf.*Table 1), even for the largest pressure achieved for the sample compressed at room temperature to *ca.* 40 GPa; see Figures S3–S4. The evolution of the crystalline phases detected in this sample well corresponds to that described earlier, and YbH_2_ remains the main hydridic phase.^47−49^ The crystalline phase, which can be attributed to a higher hydride of ytterbium, YbH_2+*x*_, is barely detected even at *ca.* 40 GPa; see Figure S3. However, the composition is variable from sample to sample, and in the case of the second DAC, the clear, although relatively weak, signals of the *P*3̅1*m* phase of YbH_2+*x*_ are visible already at 3.6 GPa, before heating of the sample; see Figure 2a. This may be attributed to a variable degree of surface oxidation, which should hamper further hydrogenation of the sample (this problem has been recently addressed using a thin Pd coating for the synthesis of yttrium superhydrides).^22^

**Table 1:** Selected Low-Angle Reflections Originating from the Distinct Phases, as Marked in Figure 2–4 and Their Lattice Parameters^a^

phase	lattice parameters	reflections
YbH_2+*x*_*P*3̅1*m*	4.9 GPa: *a* = 6.21623(13) Å, *c* = 8.8237(5) Å	a_1_-(1 0 0), a_2_-(1 0 1), a_3_-(0 0 3), and (2 1̅ 1), a_4_-(1 1 2), a_5_-(2 1̅ 4), and (3 0 0)
YbH_2+*x*_*I*4/*m*	20.6 GPa: *a* = 7.6386(2) Å, *c* = 4.8978(3) Å	b_1_-(1 0 1), b_2_-(2 0 0), b_3_-(2 1 1), b_4_-(0 0 2), and (3 1 0), b_5_-(3 1 2) and (4 2 0)
YbH_2+*x*_*I*4/*mmm*	39.2 GPa: *a* = 3.2837(8) Å, *c* = 4.6955(11) Å	c_1_-(1 0 1), c_2_-(0 0 2), and (1 1 0), c_3_-(1 1 2) and (2 0 0), c_4_-(1 0 3) and (2 1 1)
YbH_2_*P*nma	3.6 GPa: *a* = 5.7134(3) Å, *b* = 3.49242(15) Å, *c* = 6.6346(4) Å	h_1_-(1 0 1), h_2_-(0 0 2), h_3_-(0 1 1), h_4_-(1 0 2), and (2 0 0), h_5_-(1 1 1)
Yb *Fm*3̅*m*	3.6 GPa: *a* = 5.1173(4) Å	y_1_-(1 1 1), y_2_-(0 0 2)

a For more specific data, see the Supporting information.

The *in situ* laser heating of the partially hydrogenated ytterbium sample up to *ca.* 2100 K, with several pulses lasting for a few seconds each, facilitates further hydrogenation and delivers the *P*3̅1*m* phase of YbH_2+*x*_ (Figure 1a), which predominates the powder diffraction pattern; see Figures 2b and S5–S7. The YbH_2_ is detected in the sample in a minor amount, together with very weak signals from an unidentified crystalline phase(s); see Figure S7. Although the sample does remain not fully homogeneous, it can be concluded that both of these phases disappear after a few additional heating cycles on larger compression; see Figure S8. Above *ca.* 12.5 GPa, the *P*3̅1*m* phase of YbH_2+*x*_ undergoes a transition to the *I*4/*m* phase, which is isostructural to that reported for EuH_2+*x*_ at 8.7–9.7 GPa,^27^ as indicated by the peak splitting (*e.g.*, the peak b_4_ of (0 0 2) and (3 1 0) reflections, while the (1 1 2) reflection of the trigonal phase contributes solely to the related a_4_ peak) and the low-intensity reflections from the tetragonal superstructure (*e.g.*, (1 1 0), (1 0 1), and (2 0 0), marked as b_0_, b_1_, and b_2_, respectively); see Figure 2c. During further compression, the latter phase symmetrizes to the *I*4/*mmm* structure (Figure 2d), similar to the EuH_2+*x*_ analogue reported for hydrogen pressures exceeding 9.7 GPa.^27^ That second phase transition manifests itself as the vanishing superstructure signal and as a less-pronounced peak splitting for the main peaks (*e.g.*, c_2_ corresponding to (0 0 2) and (1 1 0) reflections).

**Figure 3:**
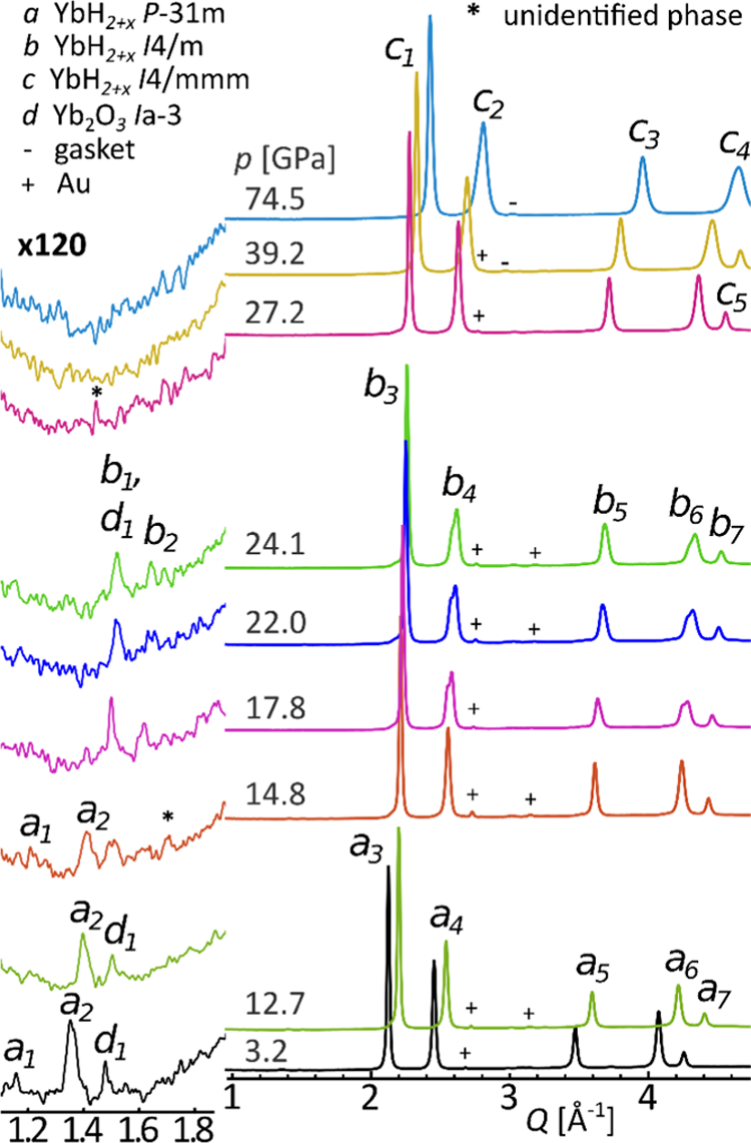
Diffraction patterns for the sample of Yb_3_H_8_ compressed in H_2_ at room temperature. The low-*Q* part has been additionally presented in 120× magnification of intensity. λ = 0.4066 Å. a-YbH_2+*x*_*P*3̅1*m*: 1-(1 0 0), 2-(1 0 1), 3-(0 0 3), and (2 -1 1), 4-(1 1 2); b-YbH_2+x_*I*4/*m*: 1-(1 0 1), 2-(2 0 0), 3-(2 1 1), 4-(0 0 2), and (3 1 0); c-YbH_2+*x*_*I*4/*mmm*: 1-(1 0 1), 2-(1 1 0), and (0 0 2).

To deal with more homogeneous samples including those with a fixed content of hydrogen, we also investigated Yb_3_H_8_ (YbH_2+*x*_, *x* = 0.67), which was prepared *ex situ* according to Auffermann’s procedure;^33^ here, Yb_3_H_8_ was studied in DAC under compression in H_2_ or in He or Ne pressure-transmitting media (PTM). The evolution of the crystalline phases for Yb_3_H_8_ compressed in H_2_ or an inert PTM well corresponds to that observed for the sample of ytterbium heated in H_2_; see Figures 3 and S10–S23. It appears that the *P*3̅1*m* and *I*4/*m* phases coexist within the range of *ca.* 13–18 GPa regardless of the PTM. The superstructure signals of the *I*4/*m* phase vanish above *ca.* 27 GPa, indicating the transition to the *I*4/*mmm* phase. However, minor low-angle diffraction peaks were still observed for some of the Yb_3_H_8_ samples compressed in the neutral PTM above 35 GPa (Figure S20). The high-symmetry *I*4/*mmm* phase of YbH_2+*x*_ remains stable up to the highest pressures reached in our investigations of *ca.* 75 GPa; see Figure 3. The first phase transition (*P*3̅1*m* → *I*4/*m*) occurs with significant changes in the first coordinating spheres and is sluggish, as both phases are detected within the *ca.* 5 GPa range (this resembles the behavior of YbH_2_).^49^ These observations suggest a reconstructive phase transition, possibly of the first-order despite no significant volume discontinuity being observed. The second phase transition (*I*4/*m* → *I*4/*mmm*) does not require significant changes in the coordination spheres but is rather related to symmetrization of the atomic positions. Therefore, it is a displacive phase transition apparently of the second order.

The transition pressure between the *I*4/*m* and *I*4/*mmm* phases of YbH_2+*x*_ is markedly higher than that of EuH_2+*x*_ containing larger metal ions.^27^ This trend remains in line with the known relations observed for the Ln^3+^ compounds, *e.g.*, following the row of contracting Ln^3+^ ions, the hexagonal-to-cubic transition in the LnH_3_ series gradually requires a higher pressure,^50,51^ and a similar trend was noticed for the phase transitions of Ln_2_O_3_.^52^ Clearly, the smaller cations require higher compression to mimic the larger ones, which is a common trend in high-pressure chemistry.^53^

**Figure 4:**
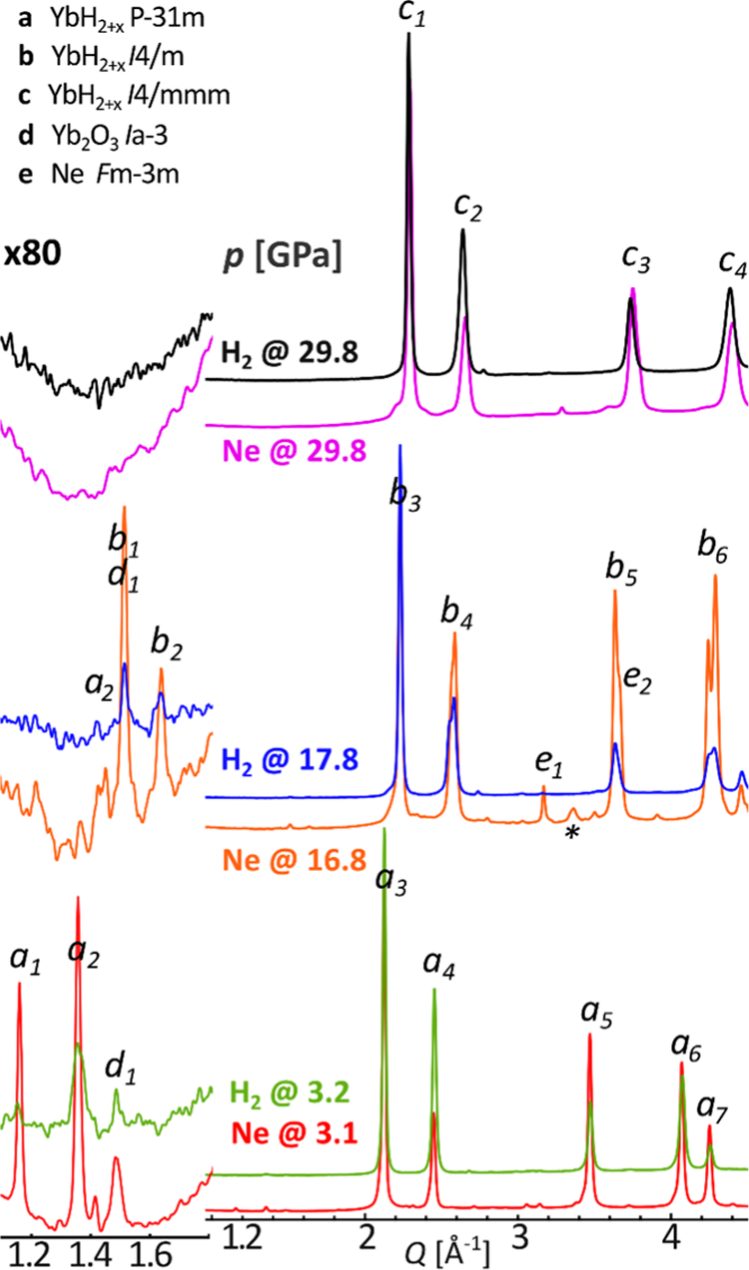
Comparison of the integrated diffraction data for Yb_3_H_8_ compressed in Ne and in H_2_. The low-*Q* region has been expanded (left). λ = 0.3344 Å (Ne at 3.1 and 16.8 GPa), 0.2952 Å (Ne at 29.8 GPa), and 0.4066 Å (H_2_). The most visible reflections of the YbH_2+*x*_ and Yb_2_O_3_ phases were marked: a-YbH_2+*x*_*P*3̅1*m*: 1-(1 0 0), 2-(1 0 1), 3-(0 0 3), and (2 1̅ 1), 4-(1 1 2); b-YbH_2+*x*_*I*4/*m*: 1-(1 0 1), 2-(2 0 0), 3-(2 1 1), 4-(0 0 2), and (3 1 0); c-YbH_2+*x*_*I*4/*mmm*: 1-(1 0 1), 2-(0 0 2), and (1 1 0), 3-(1 1 2), and (2 0 0), 4-(1 0 3), and (2 1 1). * indicates the strongest signal from the unidentified phase.

The diffraction patterns from Yb_3_H_8_ samples in H_2_ and Ar PTMs (Figure 4) are quite similar to each other. All key reflections may be assigned to the same crystalline phase in both data sets. Such a qualitative picture alone may suggest that an additional H_2_ uptake does not occur for Yb_3_H_8_ even at 74.5 GPa, the highest pressure reached in our experiments. However, for the *I*4/*mmm* phase, the corresponding diffraction signals are shifted toward lower *Q* values for the sample of Yb_3_H_8_ compressed in H_2_ PTM, indicating a larger unit cell volume. This phenomenon is noticeable around 30 GPa, becoming obvious for larger pressures (Figure S23).

**Figure 5:**
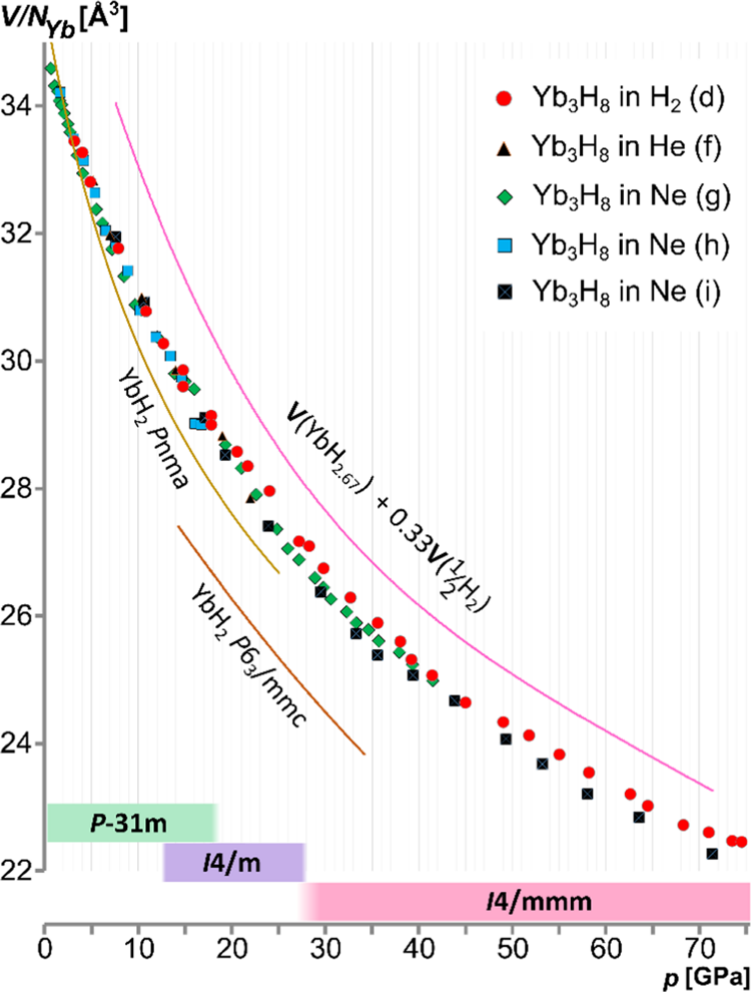
Volume per ytterbium atom in the function of pressure for the compression of Yb_3_H_8_ in H_2_, He, and Ne at room temperature. The molecular volume of YbH_2_^48^ and the volume roughly estimated for YbH_3_ have also been plotted. The crystallographic phases of YbH_2+*x*_, detected under a specific pressure range, are indicated at the bottom. Notice that the sample (d) represents a system of variable stoichiometry due to gradual H_2_ uptake.

EoS for Yb_3_H_8_ was derived independently for the YbH_2+*x*_ crystalline phases present in the samples compressed in H_2_ and Ne PTMs for the experimental runs covering a sufficient number of *V*(*p*) data points; see Figures S24–S29. The obtained fit parameters are presented in Table 2, while some of the available EoS parameters for the related lanthanide hydrides are summarized in Table S2. In general, the *V*_0_ of the low-pressure trigonal form is slightly larger than the high-pressure *I*4/*mmm* phase, and the inverse trend is observed for their bulk moduli. The *P*3̅1*m* form of Yb_3_H_8_ reveals *V*_0_ comparable to the *Pnma* YbH_2_ of *V*_0_ = 35.63(7) Å^3^, but the former remains significantly less compressible (Figure 5) (for *Pnma* YbH_2_*B*_0_ = 40.2(22) GPa).^48^ The *I*4/*mmm* phase of YbH_2+*x*_ present above *ca.* 27 GPa has a slightly smaller molecular volume with an increased bulk modulus. These values fulfill a similar trend for the europium hydrides: the *I*4/*mmm* phase of EuH_2+*x*_ of *V*_0_ = 38.8(1) Å^3^ (slightly bulkier than the Yb analogue, according to the relation of Eu and Yb ionic radii) shows a significantly higher bulk modulus in comparison to the dihydride (*B*_0_ = 69(2) GPa *vsB*_0_ = 40–45 GPa for the two polymorphs of EuH_2_).^54^ Contrastingly, the trihydrides of the neighboring lanthanides (especially the Sm for Eu and Er or Tm for Yb) reveal rather larger values of the bulk moduli, while the *V*_0_ of the *I*4/*mmm* forms of EuH_2+*x*_ and YbH_2+*x*_ falls close to the values reported for hexagonal and cubic polymorphs of the respective LnH_3_, Ln = Sm (for Eu), Er, and Tm (for Yb); see Table S2.

**Table 2:** Summary of the Parameters of the Third-Order Birch–Murnaghan EoS Obtained from the Compression Data^a^

phase of YbH_2+*x*_	PTM, sample	*V*_0_ per Yb atom [Å^3^]	*B*_0_ [GPa]	*B*_0_′	max Δ*p* [GPa]	*x*^2^, *N*
*P*3̅1*m*	Ne, (g)	35.033(32)	56.13(63)	5.5	0.31	1.82, 19
	Ne, (h)	35.31(12)	53.4(19)	5.5	–0.43	6.82, 11
	H_2_, (d)	35.292(88)	55.0(14)	5.5	0.22	3.26, 8
*I*4/*m*	Ne, (g)	35.82(23)	63.1(22)	3.0	–0.31	1.15, 8
*I*4/*mmm*	Ne, (i)	34.05(34)	64.0(33)	4.5	1.03	7.95, 10
	H_2_, (d)	34.98(27)	59.7(23)	4.5	1.67	7.21, 19

a The estimated standard deviations (E.S.Ds.) are given in parentheses. *B*_0_′ was fixed for the final refinement. *N* represents the number of data points.

One key issue is that of the composition of the YbH_2+*x*_ specimen studied and that of a possible H_2_ uptake by the sample in the H_2_ atmosphere. Inspection of Figure 5 reveals that the differences in the molar volume of the Yb_3_H_8_ specimen compressed in various PTMs are only marginal, which is also reflected by the respective EoS curves; see Figure S30. However, on larger compression, the *V*(*p*) curve seems to be slightly less steep for the sample pressurized in H_2_. Although the discrepancy is rather small and it may be caused by the errors in the determination of pressure in both types of samples, it is fairly consistent across the pressures for which the tetragonal phases are favored, especially 25–75 GPa. The EoS curves fitted for the *I*4/*mmm* YbH_2+*x*_ in H_2_ and Ne PTM fall apart by *ca.* 1.5% in terms of *V*(*p*); see Figure S31. At the same time, a simple addition of 1/3 atomic volume of hydrogen would increase the molecular volume of *I*4/*mmm* YbH_2+*x*_ by *ca.* 4.8% at this pressure range. Altogether, this might suggest that the composition of the sample compressed in excess H_2_ is similar to YbH_2.77_ rather than YbH_2.67_.

Indeed, one might speculate that the amount of 1/3 of vacancies is not a natural value for the tetragonal *I*4/*mmm* cell and that 1/4 would be a more natural value (hence, YbH_2.75_ would be a more appropriate composition for such a specimen, very close to YbH_2.77_ suggested above from the EOS analyses). However, due to lack of direct evidence supporting such a scenario, one must refrain from making any firm conclusions here. In any case, the stoichiometry of the samples studied must be still far from the one expected for the Yb(III)H_3_ stoichiometry (Figure 5, n.b. the V/N_Yb_ curve of YbH_3_ may only serve as a rough estimation as the effects of the strain introduced during crystal formation were not considered). Resistance of Yb to be fully oxidized to Yb(III) in the hydride environment is remarkable; it turns out that only if a negative charge density on the hydride is reduced, *e.g.*, by binding it to B(III) in the form of the BH_4_^–^ anion, the stoichiometric Yb(III) species may form and yet they are thermodynamically metastable at ambient (*p*,*T*) conditions.^55−58^

**Figure 6:**
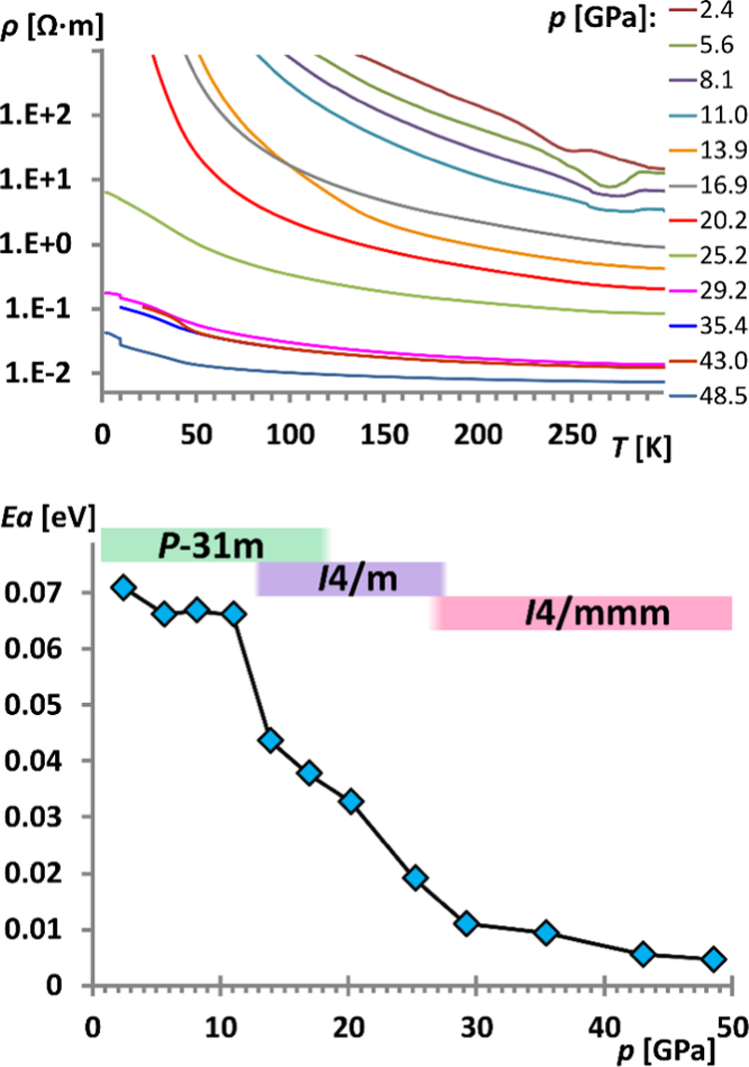
Resistivity *vs T[*K] for Yb_3_H_8_ compressed in NaCl presented in a logarithmic scale indicating a semiconductor-like behavior (top). Evolution of the activation energy of the electronic transport as a function of pressure (bottom). Despite the significant decrease of *E*_a_, the system avoids full metallization until 50 GPa (the highest pressure in our experiment).

The electric conductivity of formally mixed-valence species, be it YbH_2.67_, YbH_2.75_, or YbH_2.77_, is of great interest in the context of potential generation of superconductivity in this material. Figure 6 (top) shows the electric resistivity versus temperature plot for a sample of Yb_3_H_8_ embedded in NaCl PTM. It is clear that the low-pressure phases of Yb_3_H_8_ show a semiconducting behavior as their resistivity strongly decreases with the temperature increasing. A large drop in resistivity may be seen between 20.2 and 25.2 GPa, roughly corresponding to the second structural phase transition from the *I*4/*m* to the *I*4/*mmm* polymorphs.

Analysis of the activation energy for conductivity, *E*_a_ (Figure 6 bottom), suggests that *E*_a_ is already quite small (*ca.* 0.07 eV or 812 K) for Yb_3_H_8_ at a low pressure (2.4 GPa). This feature comes certainly from the mixed-valence nature of the compound. One sharp drop of the *E*_a_ value is seen between 11.0 and 13.9 GPa, roughly corresponding to the first crystallographic phase transition. The second drop of the *E*_a_ value between 20 and 29 GPa is more subtle, which suggests that the second phase transition affects *E*_a_ less considerably, and it is followed by the flattening of the curve above *ca.* 29 GPa. The *E*_a_ value determined at 48.5 GPa is as small as 0.005 eV (58 K) yet not null. Thus, Yb_3_H_8_ retains its semiconducting character even at *ca.* 50 GPa; this makes it similar to Fe_3_O_4_.^59^ Metallicity, which would correspond to the formulation of Yb(III)_3_H_8_(e^–^), might be within the reach of the 1 Mbar experiments.

To learn what properties might be expected from fully stoichiometric YbH_3_, we performed quantum mechanical calculations for this compound; we assumed that it would adopt the *I*4/*mmm* structure observed here, similar to the europium analogue for which solely the Eu^3+^ oxidation state has been detected above 12.5 GPa.^28^ Both the magnetic and spin-unpolarized models were considered. The molecular volumes calculated for these models of YbH_3_ fall within the range of experimentally observed values for the YbH_2.67_ and YbH_2.67+*x*_ compounds (Figure S33), which may indicate a slight underestimation of the lattice parameters by the applied DFT formalism. At the same time, the molecular volume computed for the YbH_4_ polyhydride (*I*4/*mmm*, isostructural with the proposed YH_4_ and other REH_4_)^60^ remains significantly larger.

**Figure 7:**
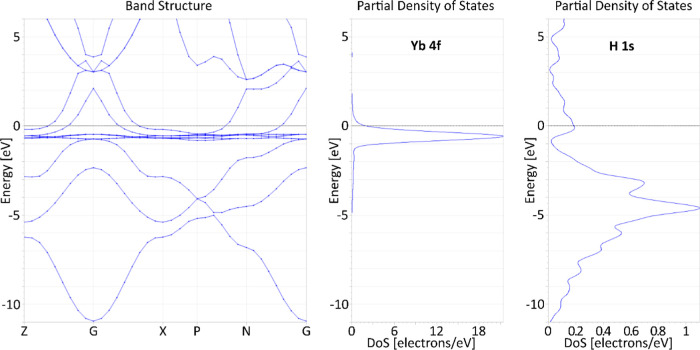
Electronic band structure (left) and DOS (Yb 4f center, H 1s right) for the spin-unpolarized solution, for YbH_3_ in the *I*4/*mmm* structure type at 50 GPa. Note the partial DOS scale difference between those for Yb and H electrons.

The DFT calculations for all magnetic states converge to solutions characterized by extremely small remnant magnetic moments. The corresponding spin-nonpolarized band structure and DOS (Figure 7) suggest that the compound would have a metallic nature, with several broad bands crossing the Fermi level. Both Yb(f) and H(s) states contribute to the states at the Fermi level; such strong hybridization was noticed previously.^34^ The fact that the spin of Yb(III) would not localize but instead give rise to itinerant electrons may seem surprising, but this could be due to strong hybridization with broad H(s) states (and this was the reason why we were targeting YbH_3_ composition in experiments).

## Conclusions

We have attempted synthesis of the yet-elusive ytterbium trihydride utilizing both metallic Yb and its currently most hydrogen-rich hydride, YbH_2.67_, as the precursors in high-pressure hydrogenation (up to *ca.* 75 GPa). Our results point to the lack of a marked qualitative difference between these systems compressed in H_2_ and the samples of YbH_2.67_ pressurized in He or Ne PTM. In all of these cases, a sequence of phase transitions from the unit cells of the *P*3̅1*m* symmetry to the *I*4/*m* and *I*4/*mmm* systems occurred within *ca.* 13–18 GPa and around 27 GPa, respectively. The same tetragonal phases were observed upon compression of EuH_2_ in H_2_ PTM: the *I*4/*m* Eu^2+^/Eu^3+^ mixed-valence system formed from the *P*6_3_/*mmc* dihydride above 8.7 GPa and the high-symmetry *I*4/*mmm* phase was detected above 9.7 GPa.^27^ The fact that Eu^3+^ in a hydride environment forms at a pressure as large as 8.7 GPa, while analogous species of Yb^3+^ may be prepared at several kPa, is following the standard redox potentials of the Eu^3+^/Eu^2+^ and Yb^3+^/Yb^2+^ pairs, which are −0.35 and −1.05 V, respectively. In other words, Yb^2+^ can be easier oxidized by H_2_ to Yb^3+^ than its Eu^2+^ analogue.

For the highest pressure investigated here (25–75 GPa) corresponding to the *I*4/*mmm* phase, the molecular volume of the systems compressed in H_2_ PTM consistently remains *ca.* 1.5% larger than that for the systems compressed in inert gas media. Such a difference in the molecular volume indicates incremental hydrogenation with the possible formation of YbH_2.75–2.77_ at *ca.* 75 GPa. However, additional research is necessary to fully identify the stoichiometry achieved upon hydrogenation, and an even rather larger pressure is needed to push the system toward stoichiometric YbH_3_ or ytterbium polyhydrides. Such compounds should be interesting due to their metallic nature, as expected on the basis of DFT calculations for YbH_3_. At the same time, while the mixed-valence Yb_3_H_8_ retains its semiconducting character up to at least 50 GPa, the very low remnant activation energy of conduction (<5 meV) suggests that the metallization under further compression of this mixed-valence compound should also be achievable.

## Supporting Information Available

Raw experimental data, additional figures and plots, profile and structure refinement results; CIF files—full structural details (PDF)

## Accession Codes

CCDC 2150168–2150171 contain the supplementary crystallographic data for this paper. These data can be obtained free of charge via www.ccdc.cam.ac.uk/data_request/cif, or by emailing data_request@ccdc.cam.ac.uk, or by contacting The Cambridge Crystallographic Data Centre, 12 Union Road, Cambridge CB2 1EZ, UK; fax: +44 1223 336033.
