# Supplementary material for: Synthesis, Structure, and Electric Conductivity of Higher Hydrides of Ytterbium at High Pressure
Source: Inorg Chem. 2022 Jun 1;61(23):8694–702. doi: 10.1021/acs.inorgchem.2c00405 (PMC9490838; doi:10.1021/acs.inorgchem.2c00405)
Supplement: Supplementary file 1 — ic2c00405_si_001.pdf [file ic2c00405_si_001.pdf]

Supplementary Information

Synthesis, structure and electric conductivity of higher hydrides of ytterbium at high pressure

Tomasz Jaroń,[a,b,c*] Jianjun Ying,[b,d] Marek Tkacz,[e] Adam Grzelak,[a] Vitali B. Prakapenka,[f] Viktor. V. Struzhkin[b,g*], Wojciech Grochala[a*]

[a]Centre of New Technologies, University of Warsaw, Banacha 2c, 02-097 Warsaw, Poland, [b]Geophysical Laboratory, Carnegie Institution of Washington, 5251 Broad Branch Road NW, Washington, DC 20015, USA, [c]Faculty of Chemistry, University of Warsaw, Pasteura 1, 02-089 Warsaw, Poland, [d]HPCAT, Geophysical Laboratory, Carnegie Institution of Washington, Argonne, IL 60439, USA, [e]Institute for Physical Chemistry, Polish Academy of Science, Warsaw, Poland, [f]Consortium for Advanced Radiation Sources, The University of Chicago, Chicago, IL 60637, USA, [g]Center for High Pressure Science and Technology Advanced Research, Shanghai 201203, China.

*e-mail: tjaron@uw.edu.pl, viktor.struzhkin@hpstar.ac.cn, w.grochala@cent.uw.edu.pl

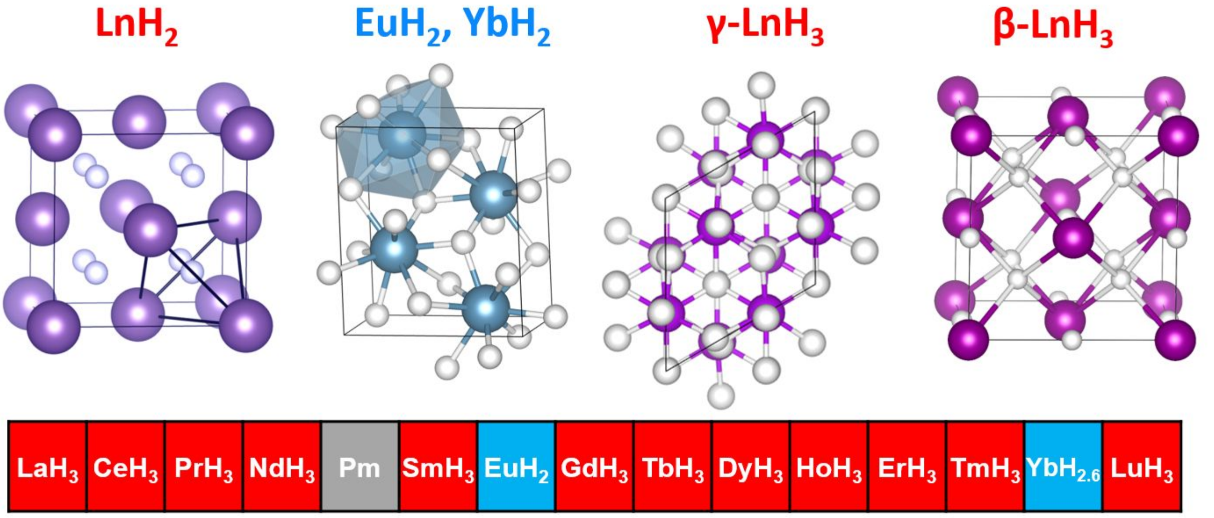

Figure S1. The crystal structures adopted by lanthanide hydrides near ambient pressure. EuH_2 and YbH_2 of ionic character crystallize in $PbCl_2$ structure (Pnma), while the other lanthanide dihydrides (metallic) – in fcc CaF_2 structure. The early LnH_3 crystallize in an fcc structure of LnH_2, but with filled octahedral sites, while the later LnH_3 – in an hcp structure of HoH_3 type. The stoichiometry of LnH_x achievable at the pressures up to ca. 0.3 GPa has been listed at the bottom.

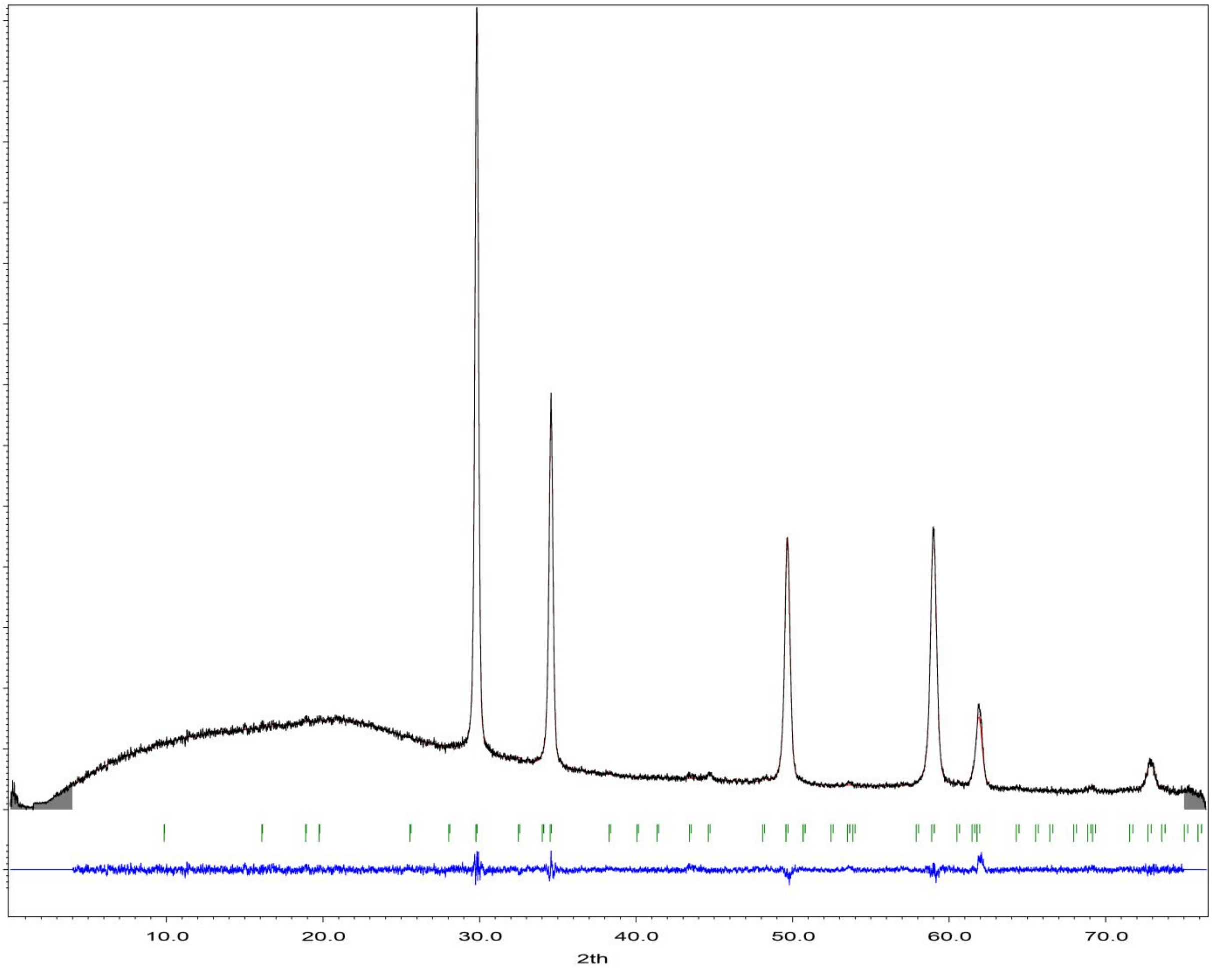

Figure S2. The PXD pattern of Yb_3H_8 measured under ambient conditions. Black curve – measured data, red – calculated, bottom – difference plot (Rietveld refinement). Cu Kα radiation has been used (λ≈1.5406 Å).

Table S1. The results of Rietveld refinement of ***Yb_3H_8*** *measured under ambient conditions.*

p [atm]	1			
T [°C]	RT, *ca. 25*			
space group	*P*-31m (162)			
a [Å]	6.3699(18)			
c [Å]	9.007(5)			
V [Å^3]	316.50(10)			
3Z	9			
V/3Z [Å^3]	35.167(11)			
wRp; cwRp [%]	4.00; 10.51			
d_{calc} [g cm^{-3}]	8.298(3)			
Yb1	0	0	0	Biso 2.39(6)*
Yb2	1/3	2/3	0	Biso 2.39(6)*
Yb3	0.3484(5)	0	0.3298(10)	Biso 2.39(6)*
H1	0	0	0.644	Biso 3**
H2	1/3	2/3	0.219	Biso 3**
H3	0.322	0	0.5812	Biso 3**
H4	0.356	0	0.0757	Biso 3**
H5	0.2364	0	0.8319	Biso 3**

* Uiso of all Yb atoms has been set as equal, ** the parameters of H atoms were not refined

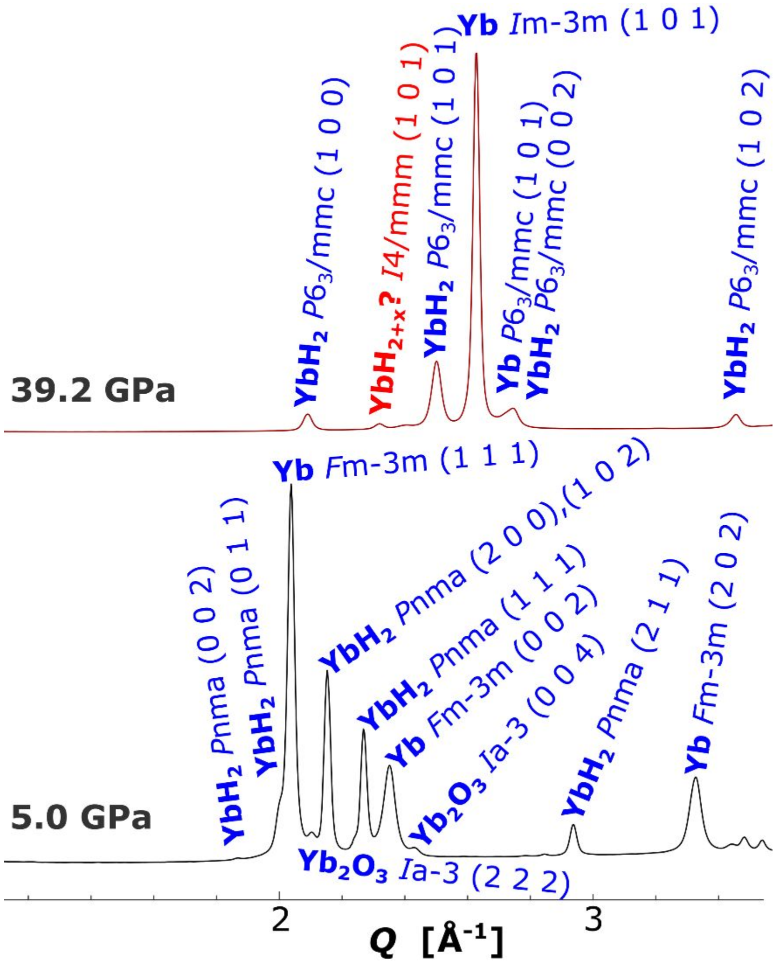

Figure S3. The diffraction data for the products of the reaction between Yb and H_2 performed at room temperature: p = 5.0 GPa and 39.2 GPa. The low-Q area has only been shown for better visibility. λ = 0.3344 Å. The most visible signal possibly originating from the higher ytterbium hydride has been marked with the red font. YbH_{2+x}: I4/mmm, a ≈ 3.307 Å, c ≈ 4.709 Å, V ≈ 51.49 $Å^3$.

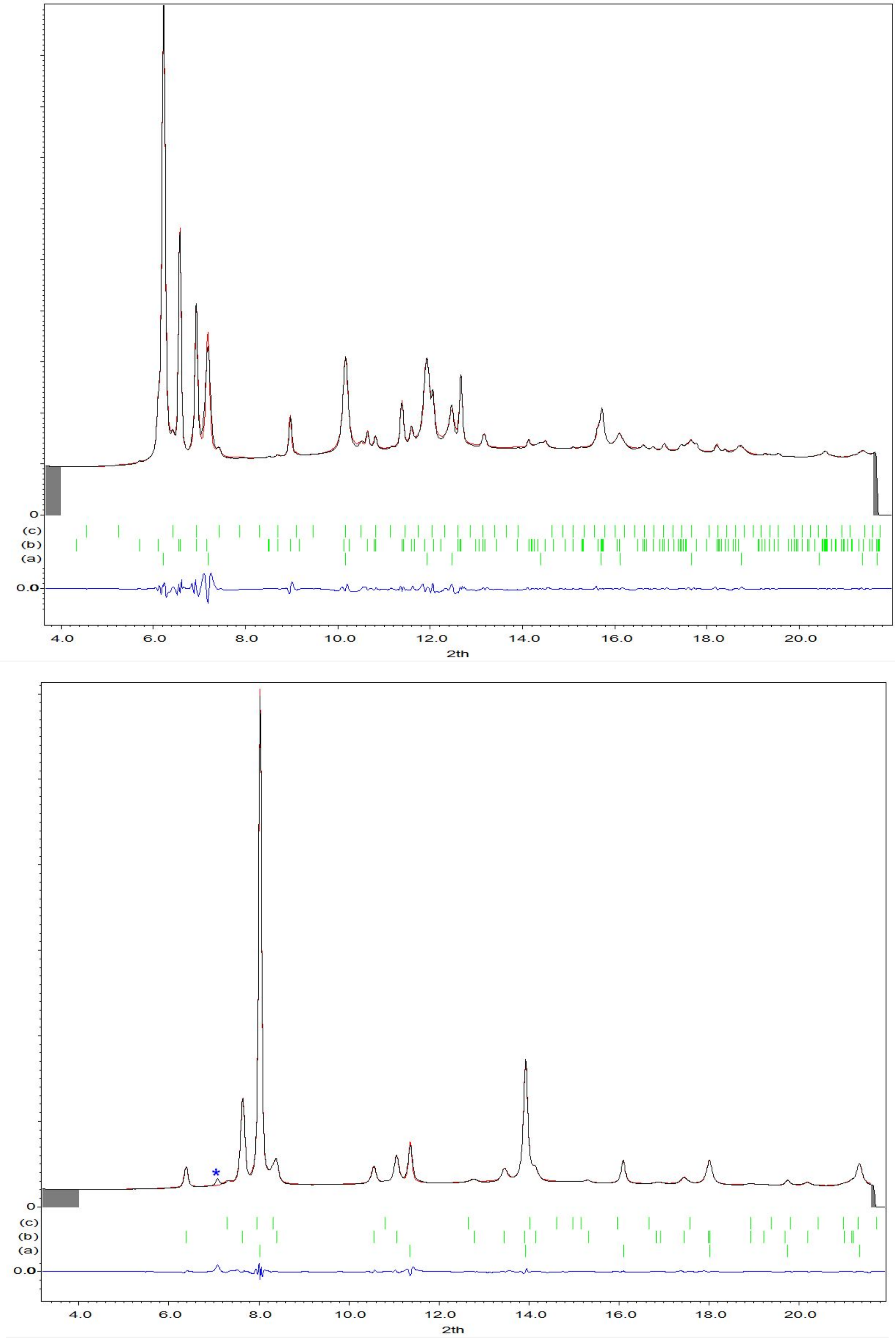

*Figure S4. The typical diffraction data for the products of the reaction between Yb and H_2 performed at room temperature. **Top**: p = 5.0 GPa, (a) – Yb Fm-3m, (b) – YbH_2 Pnma, (c) – Yb_2O_3 Ia-3. **Bottom**: p = 39.2 GPa, (a) – Yb Im-3m, (b) – YbH_2 $P6_3/mmc$, (c) – Yb $P6_3/mmc$, * – (1 0 1) reflection of YbH_{2+x} I4/mmm. LeBail fit has been marked with a red line, while the positions of Bragg reflections and the difference curve have been plotted at the bottom. λ = 0.3344 Å.*

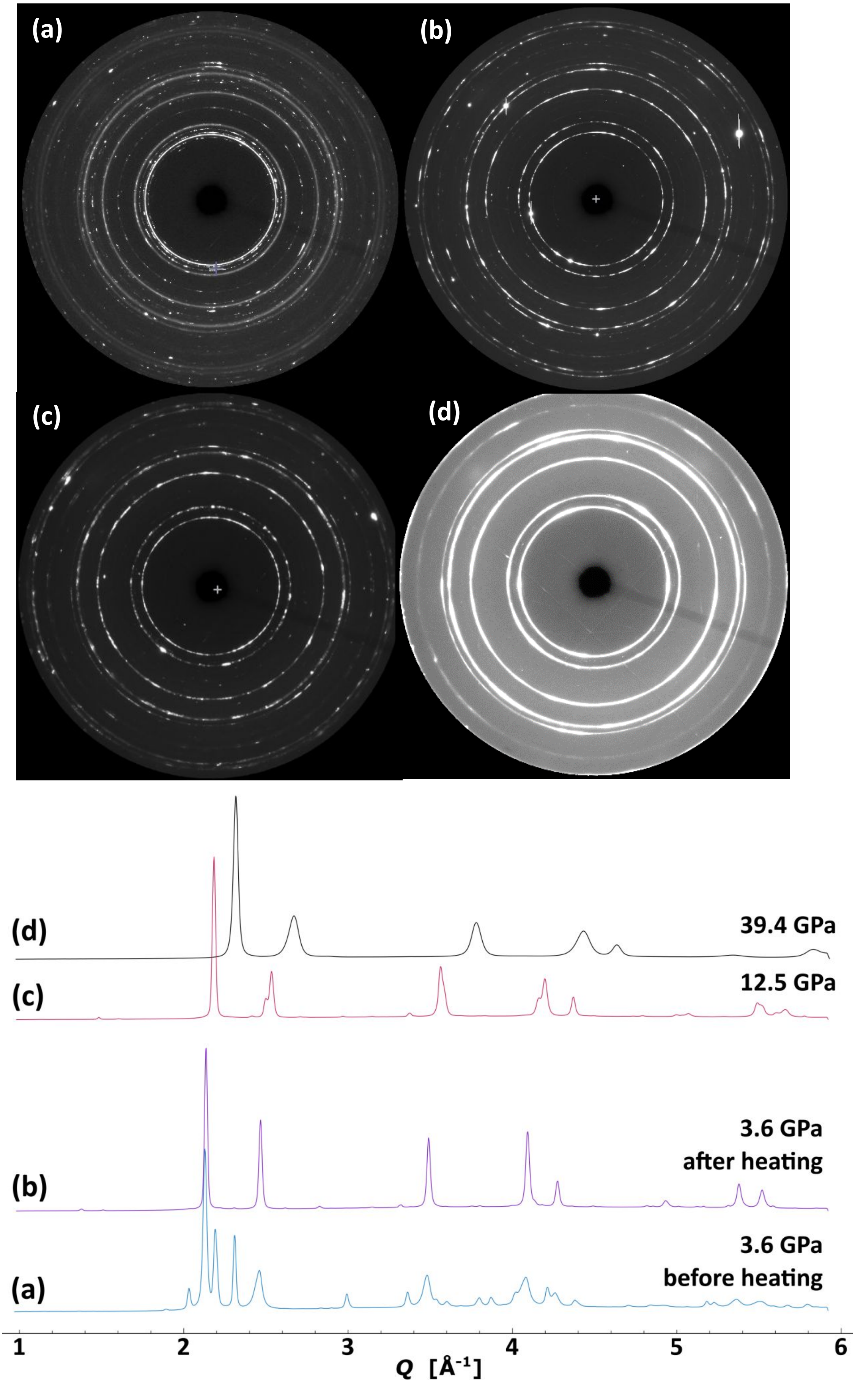

Figure S5. The raw diffraction images for the sample of Yb compressed in H_2 (top), and the corresponding integrated diffraction patterns: (a) before and (b) after the laser heating at ca. 3.6 GPa; (c) after several rounds of laser heating (ca. 12.5 GPa); (d) at ca. 39.4 GPa. Notice the simplification of the pattern (due to disappearance of YbH_2 and Yb phases) and better averaging of the diffraction signals after heating. λ = 0.3344 Å.

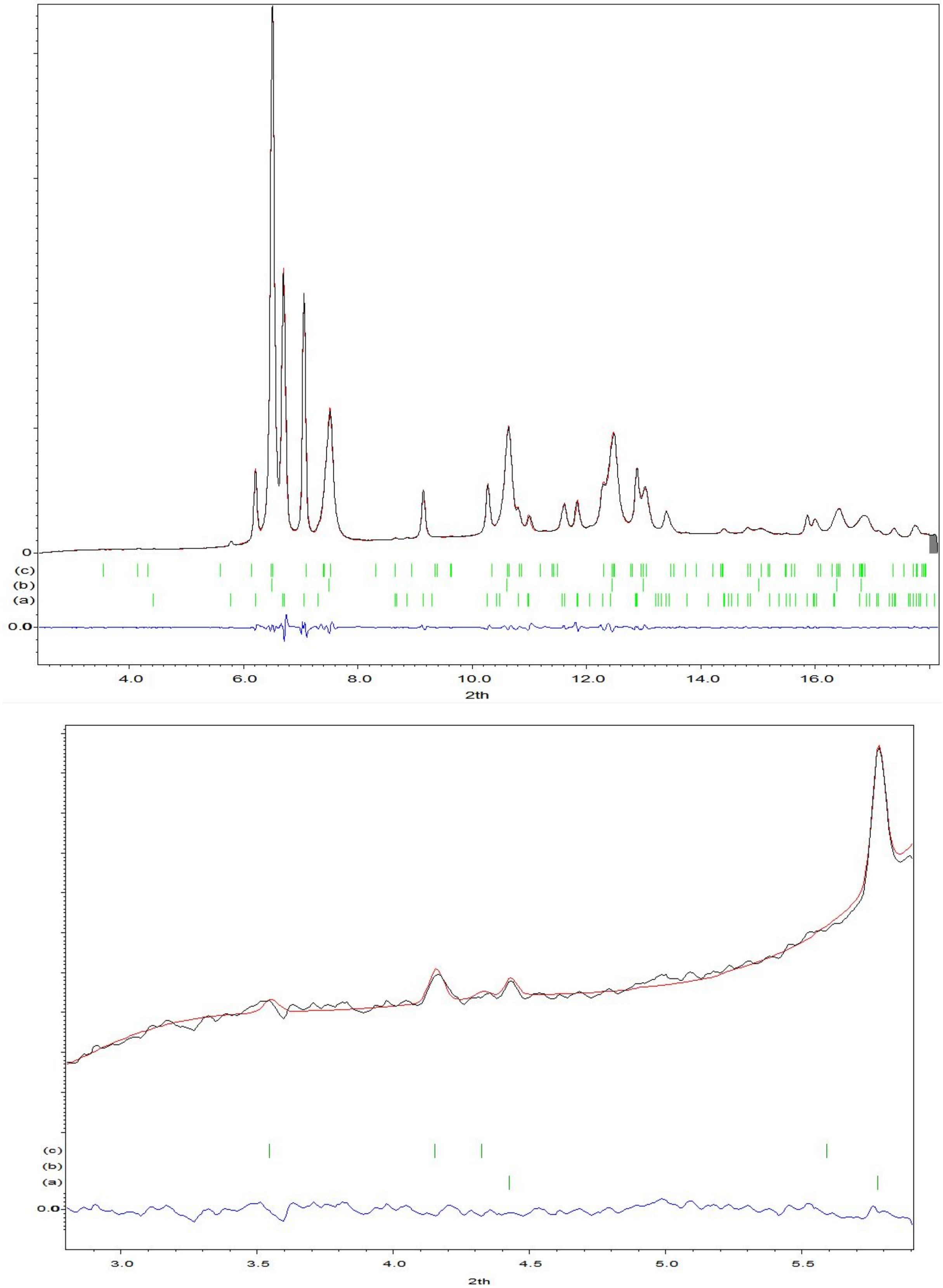

Figure S6. The diffraction data for the products of the reaction between Yb and H_2 before the system was heated. ***p*** *= 3.6 GPa, (a) – YbH_2 Pnma, (b) – Yb Fm-3m, (c) – YbH_{2+x} P-31m. LeBail fit has been marked with a red line, while the positions of Bragg reflections and the difference curve have been plotted at the bottom. The low-angle region has been shown at the bottom, revealing i.a. a weak (1 0 1) reflection of the YbH_{2+x} P-31m phase (ca. 4.15^o). λ = 0.3344 Å.*

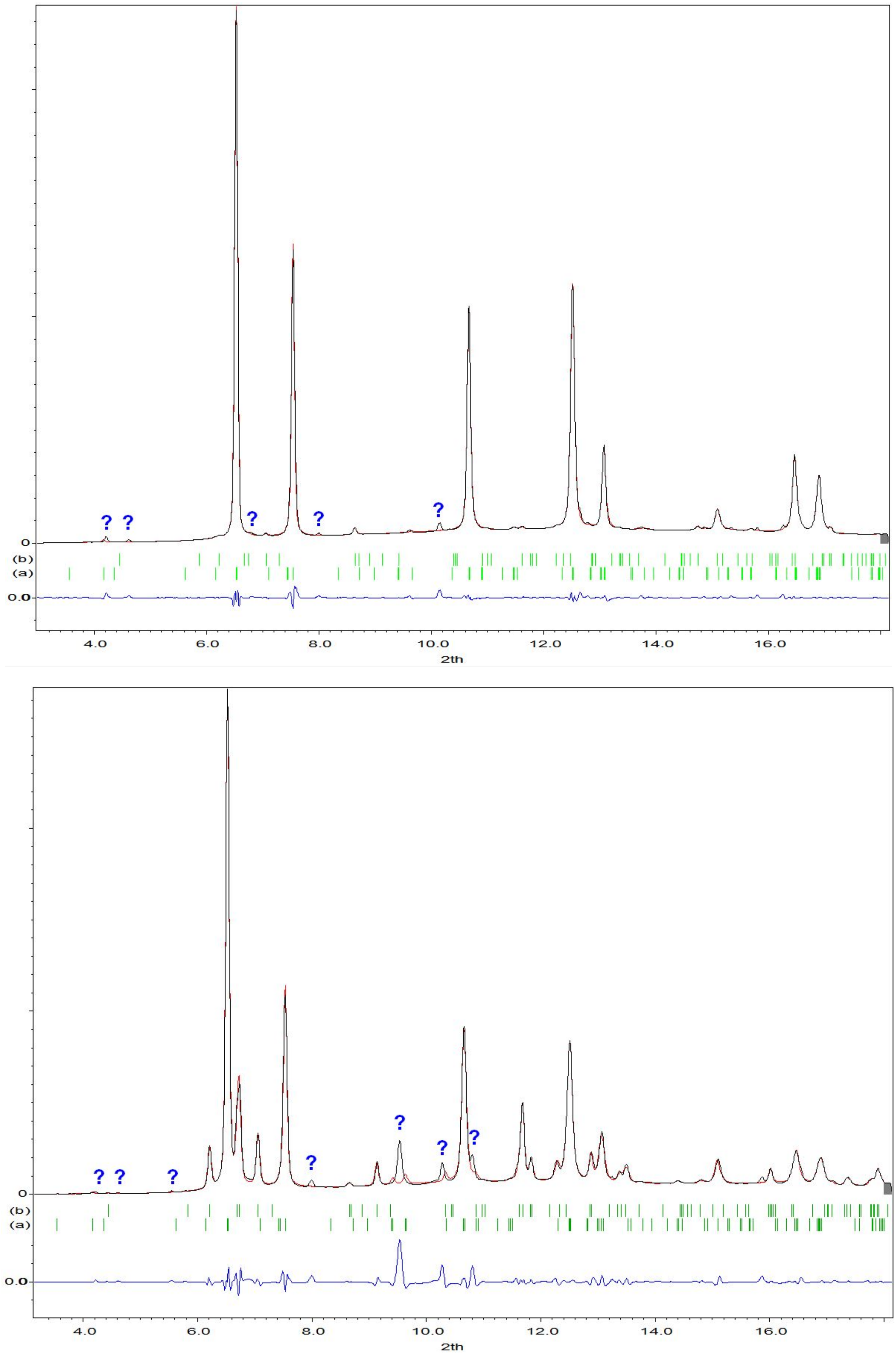

Figure S7. The diffraction data for the products of the reaction between Yb and H_2 after laser heating at ca. 3.6 GPa, as measured in the two areas of the sample (the top and the bottom plots): (a) – YbH_{2+x} P-31m, (b) – YbH_2 Pnma. LeBail fit has been marked with a red line, while the positions of Bragg reflections and the difference curve have been plotted at the bottom. The signals from the unidentified phase(s) have been marked with a "?". λ = 0.3344 Å. Note the variable contribution of the crystalline phases across the sample.

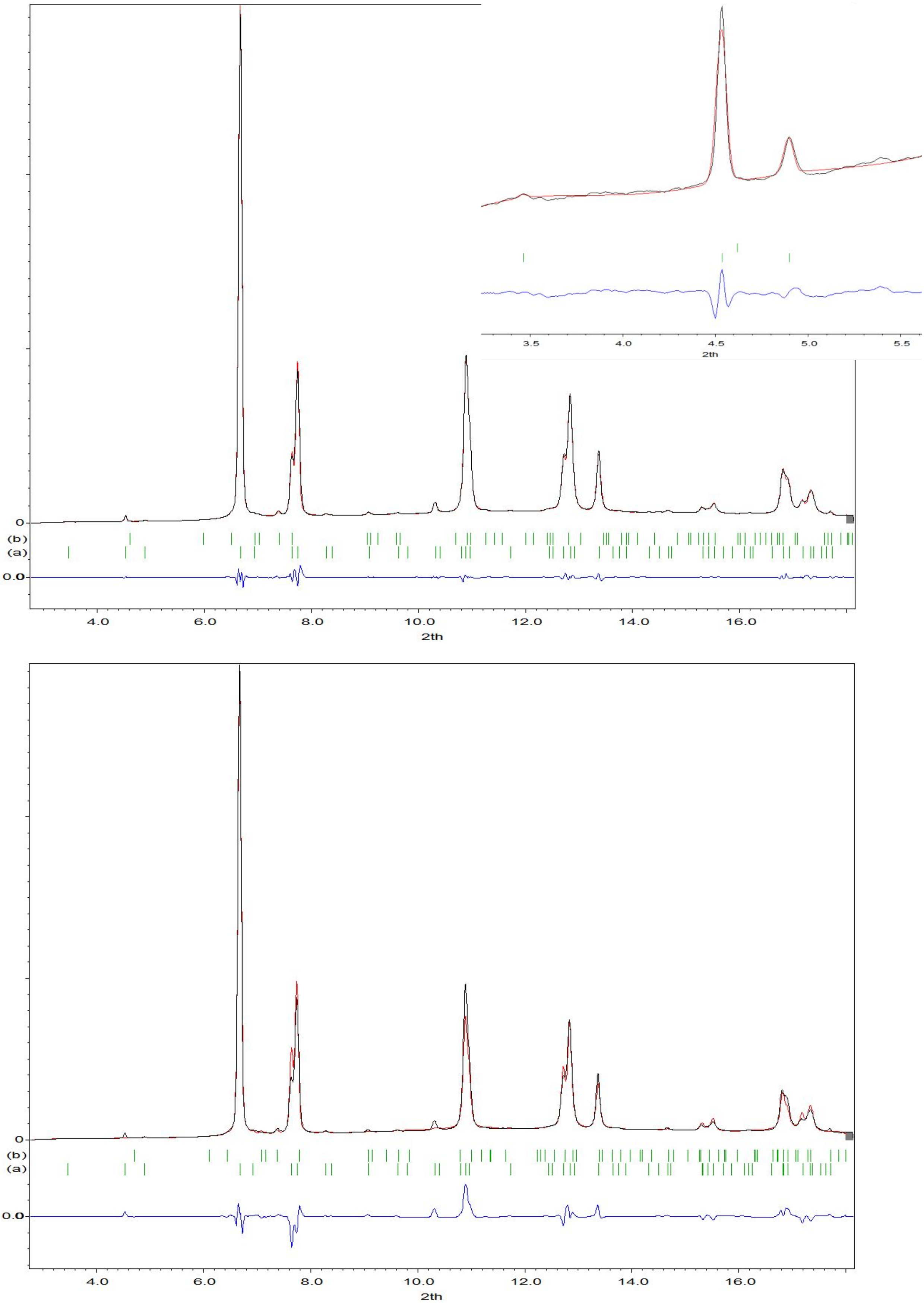

Figure S8. The diffraction data for the products of the reaction between Yb and H_2 after several cycles of laser heating, compressed to ca. 12.5 GPa. Top – LeBail fit, bottom – ***Rietveld*** *fit with no correction for the preferred orientation: (a) – YbH_{2+x} P4/m, (b) – YbH_2 Pnma. The fit has been marked with a red line, while the positions of Bragg reflections and the difference curve have been plotted at the bottom. λ = 0.3344 Å.*

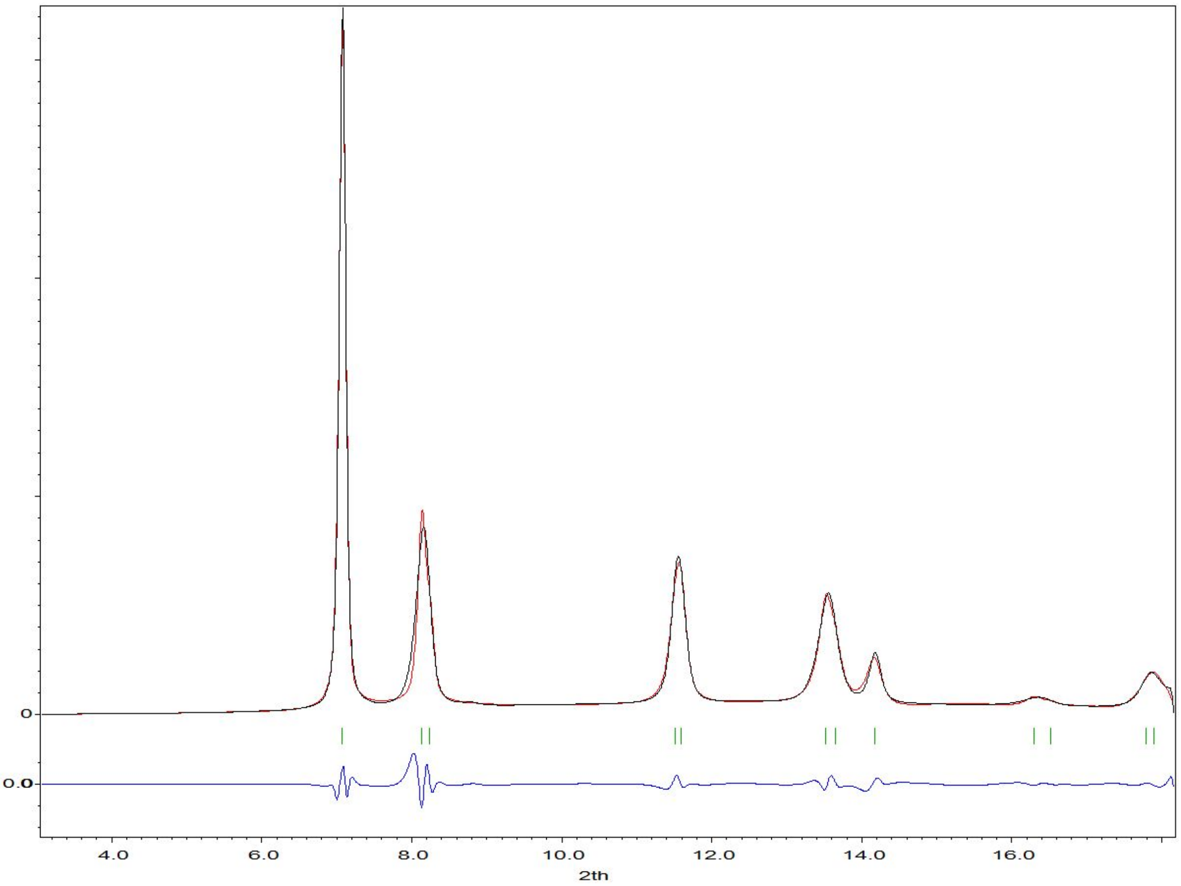

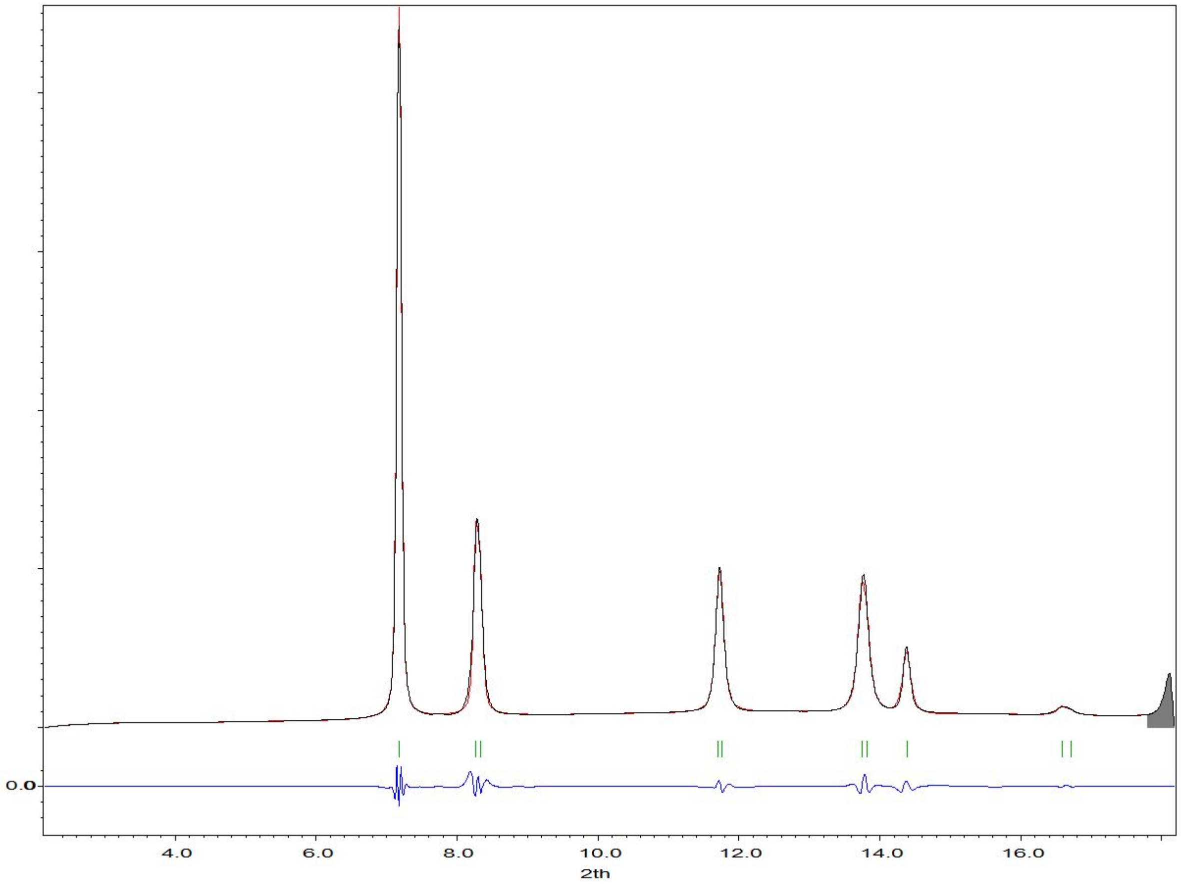

Figure S9. The diffraction data for the products of the reaction between Yb and H_2 after several cycles of laser heating, compressed to ca. 39.4 GPa, as measured in the two areas of the sample: YbH_{2+x} P4/mmm. The LeBail fit has been marked with a red line, while the positions of Bragg reflections and the difference curve have been plotted at the bottom. λ = 0.3344

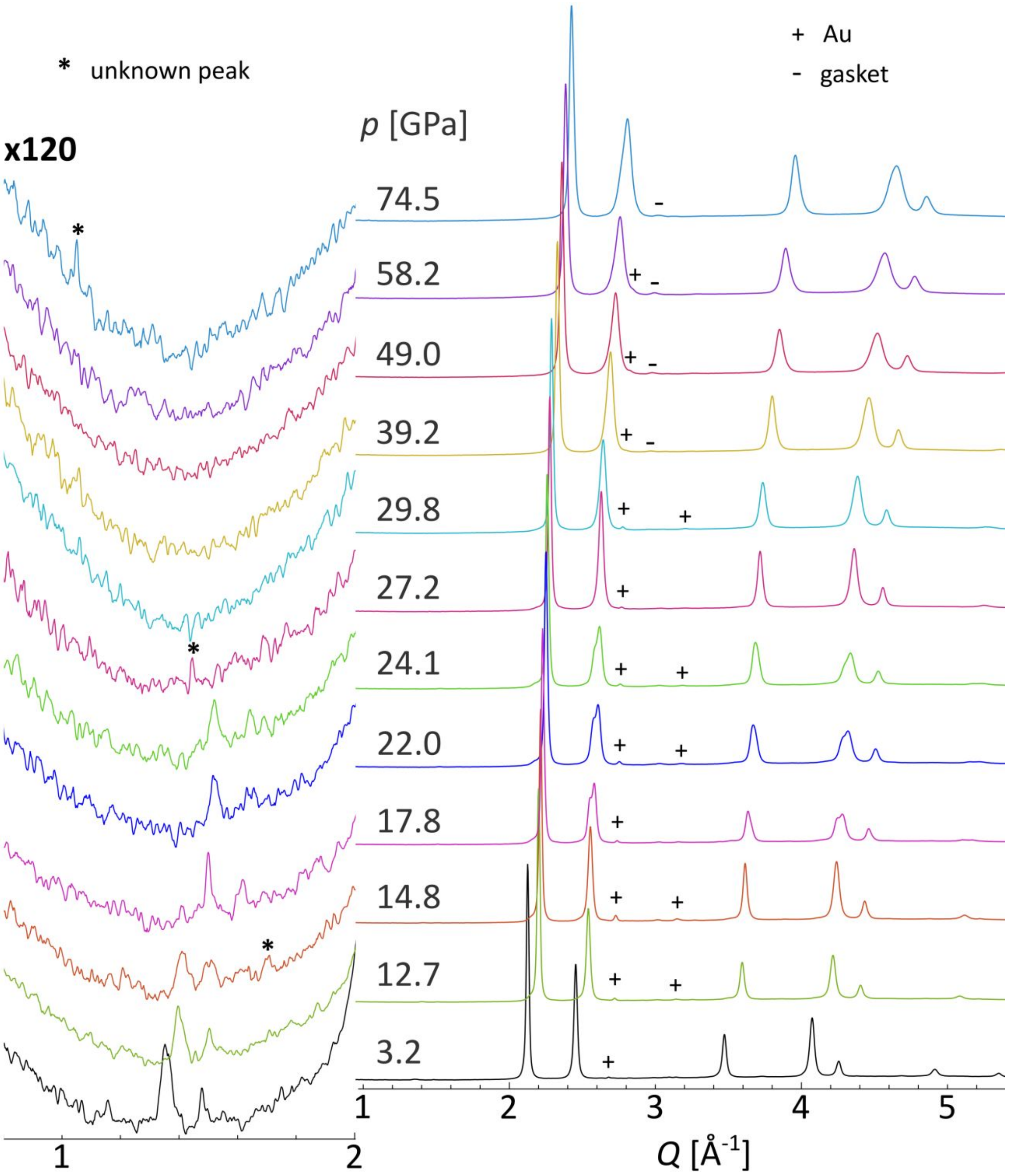

Figure S10. The integrated diffraction data for Yb_3H_8 compressed in H_2 (right) with the expanded low-Q region (left). λ = 0.4066 Å.

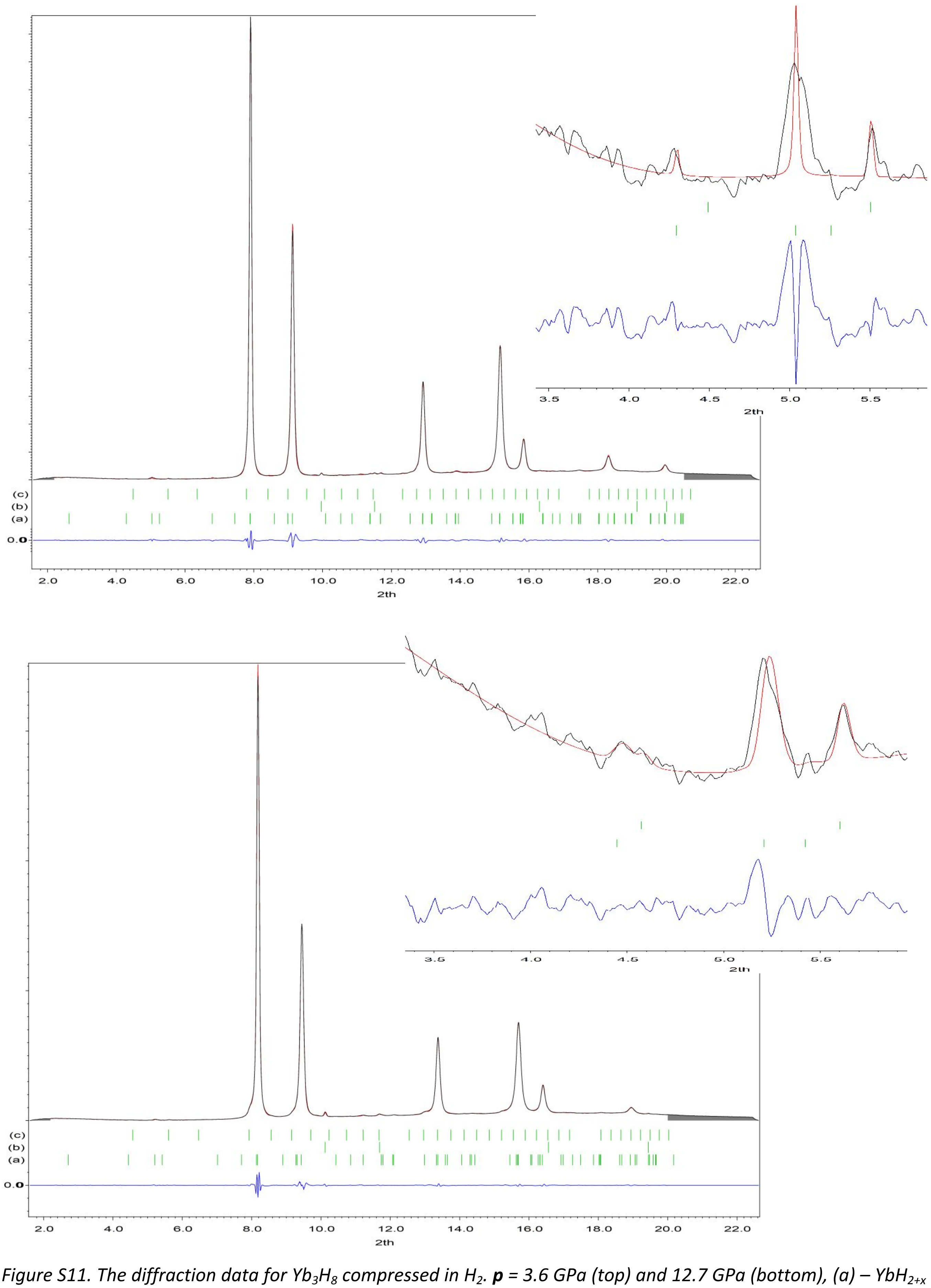

*Figure S11. The diffraction data for Yb_3H_8 compressed in H_2. **p** = 3.6 GPa (top) and 12.7 GPa (bottom), (a) – YbH_{2+x} P-31m, (b) – Au Fm-3m, (c) – Yb_2O_3 Ia-3. LeBail fit has been marked with a red line, while the positions of Bragg reflections and the difference curve have been plotted at the bottom. The low-angle area is shown as an inset. λ = 0.4066 Å.*

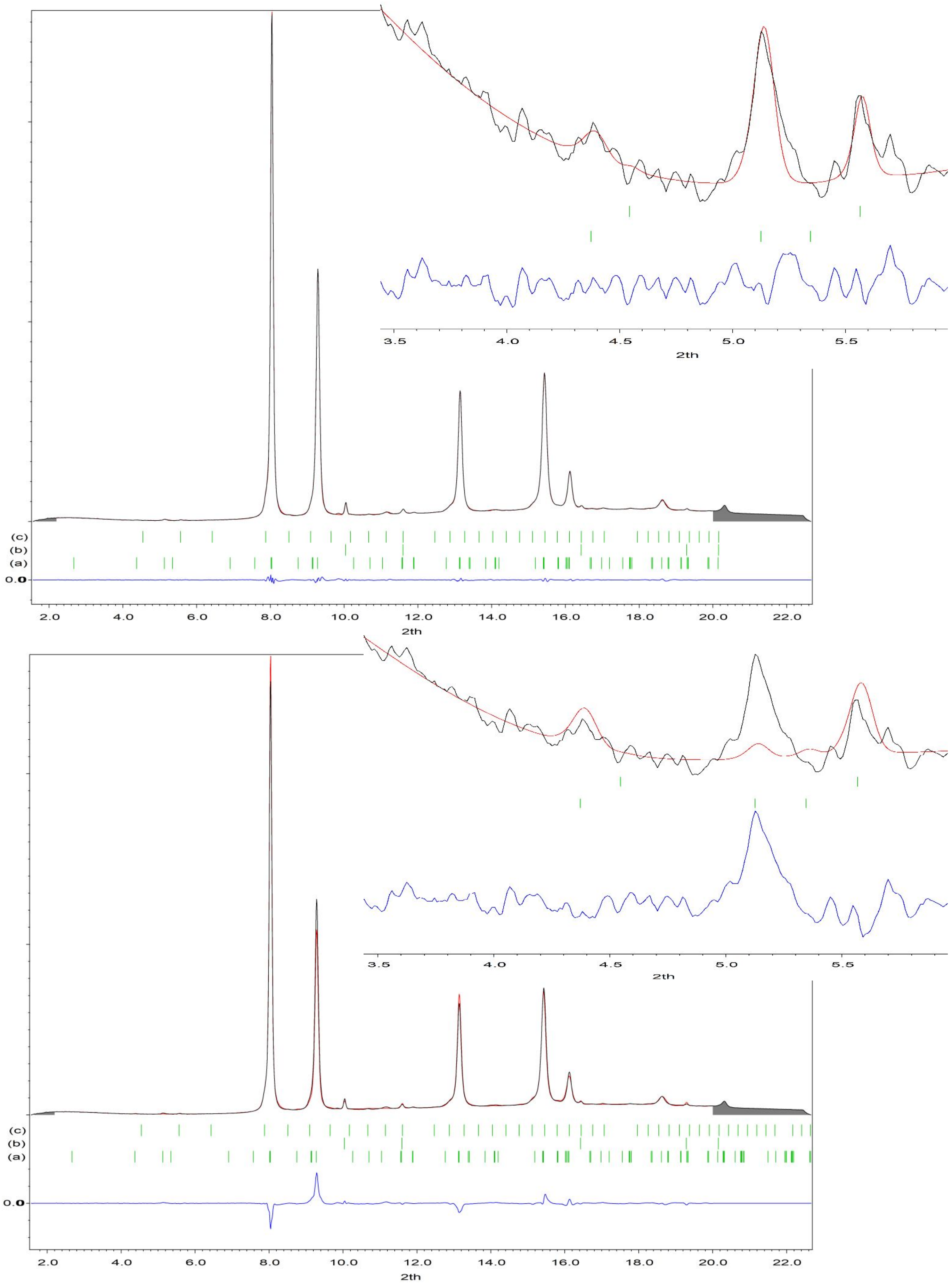

*Figure S12. A comparison of the LeBail (top) and **Rietveld** fits (bottom) for Yb_3H_8 compressed in H_2. **p** = 7.9 GPa, (a) – YbH_{2+x} P-31m, (b) – Au Fm-3m, (c) – Yb_2O_3 Ia-3. LeBail (top plot) and Rietveld (bottom plot) fit have been marked with a red line, while the positions of Bragg reflections and the difference curve have been plotted at the bottom. The low-angle area is shown as an inset. λ = 0.4066 Å. The relative phase amounts in wt.%: YbH_{2+x} (as Yb_3H_8): 96.6(9); Au: 1.02(16); Yb_2O_3: 2.9(9). LeBail fit: wRp = 2.28%. Rietveld fit: wRp = 7.30%.*

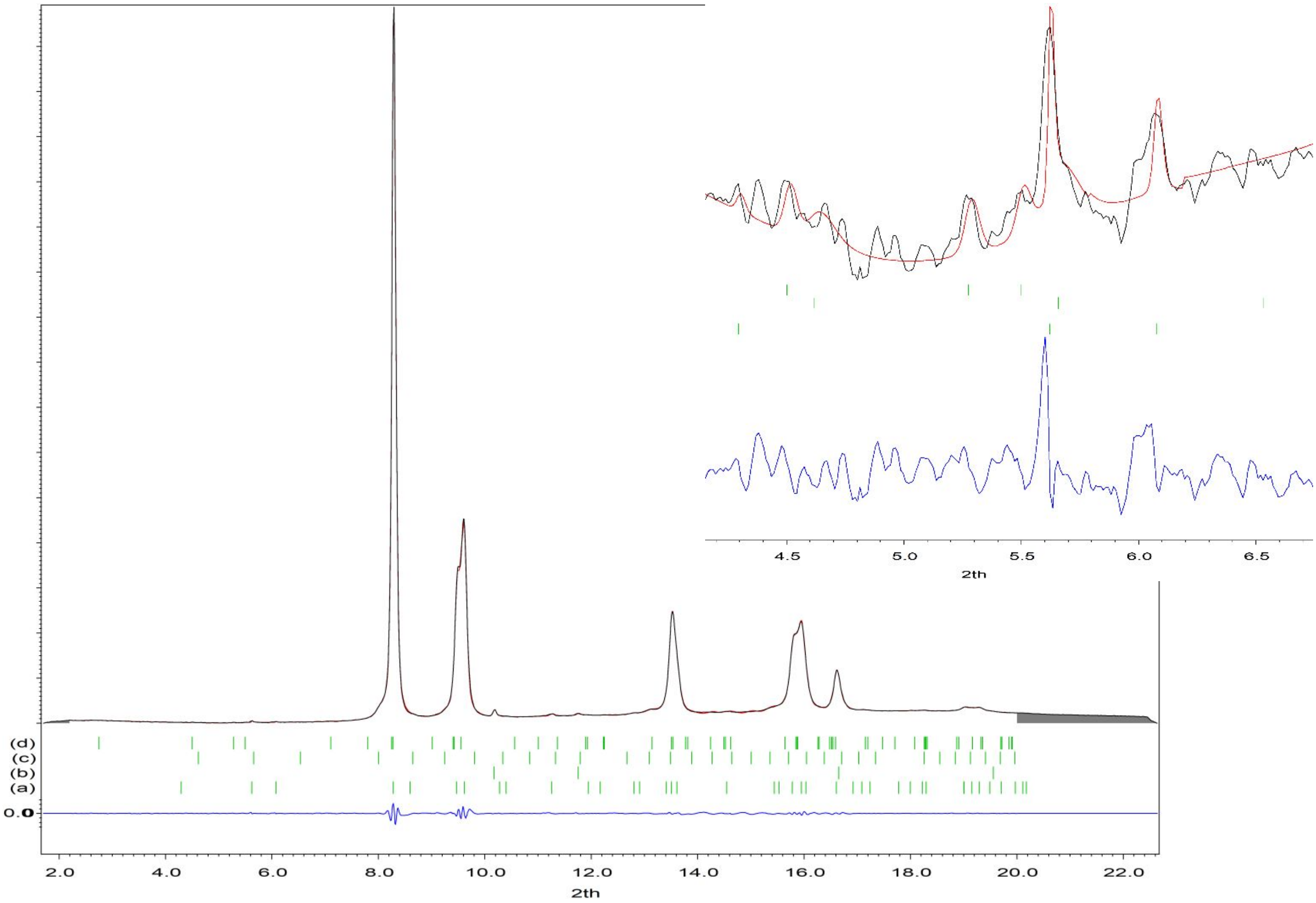

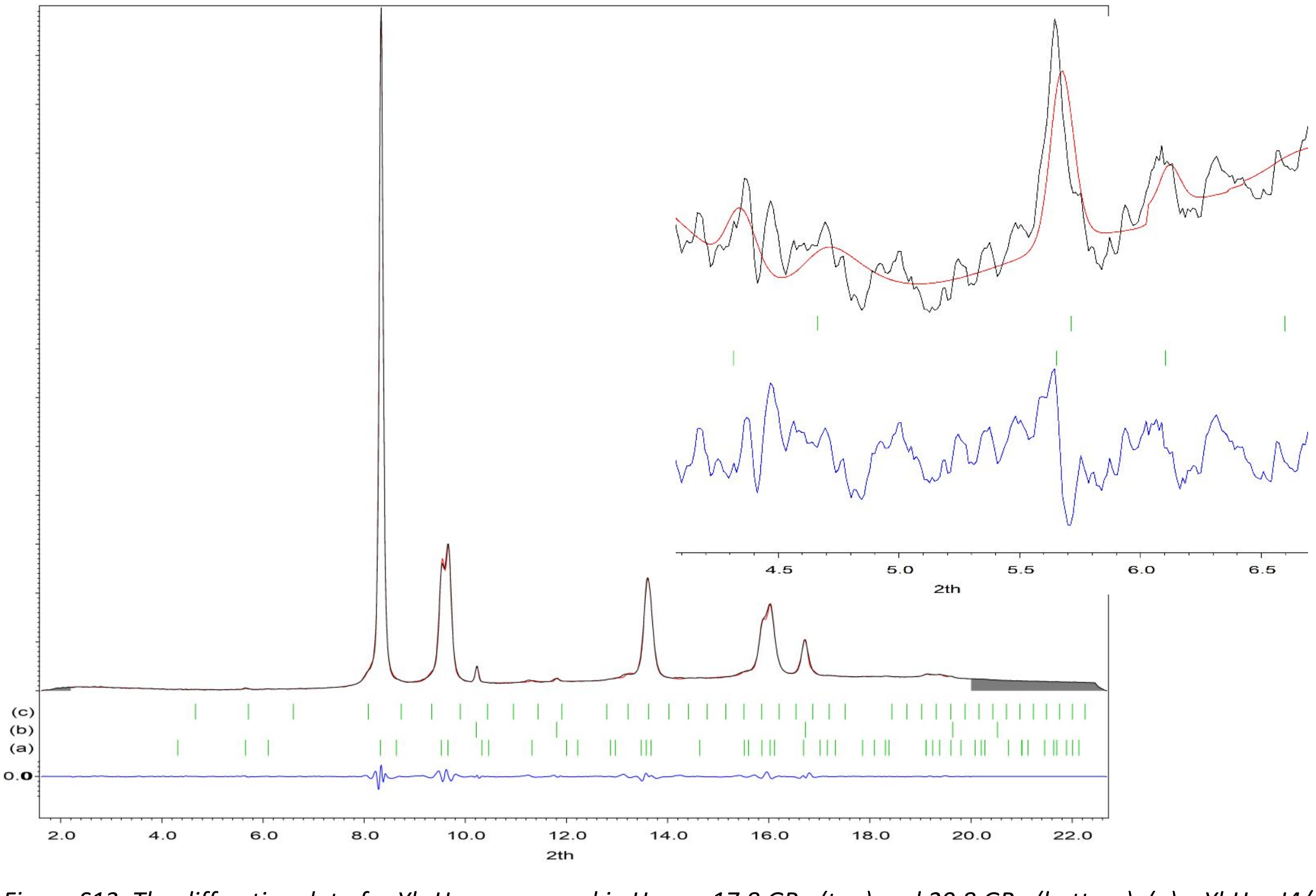

*Figure S13. The diffraction data for Yb_3H_8 compressed in H_2. **p** = 17.8 GPa (top) and 20.8 GPa (bottom), (a) – YbH_{2+x} I4/m, (b) – Au Fm-3m, (c) – Yb_2O_3 Ia-3, (d) – YbH_{2+x} P-31m. LeBail fit has been marked with a red line, while the positions of Bragg reflections and the difference curve have been plotted at the bottom. The low-angle area is shown as an inset. λ = 0.4066 Å.*

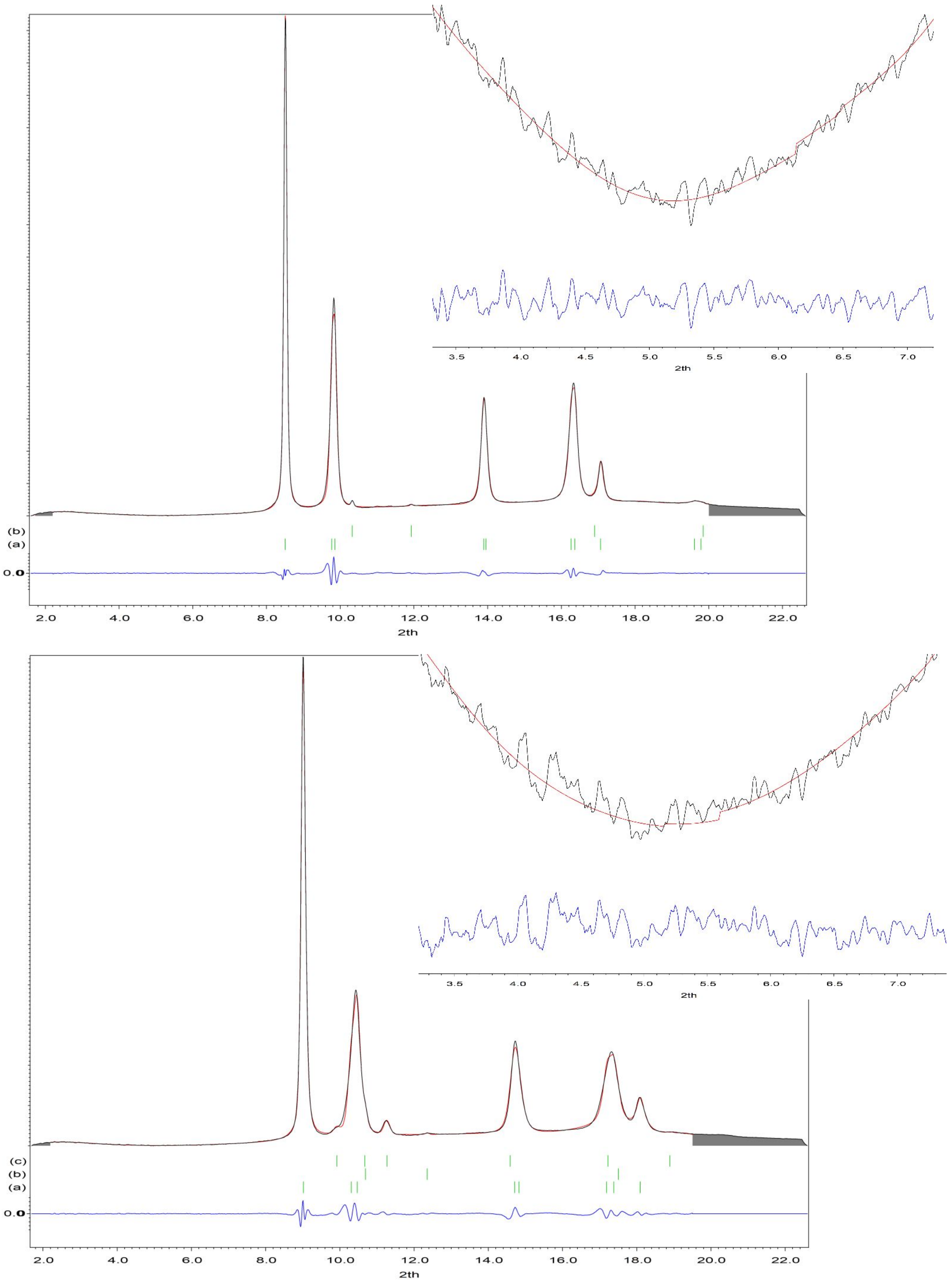

*Figure S14. The diffraction data for Yb_3H_8 compressed in H_2. **p** = 29.8 GPa (top) and 73.5 GPa (bottom), (a) – YbH_{2+x} I4/mmm, (b) – Au Fm-3m, (c) – Re gasket $P6_3/mmc$. LeBail fit has been marked with a red line, while the positions of Bragg reflections and the difference curve have been plotted at the bottom. The low-angle area is shown as an inset. λ = 0.4066 Å.*

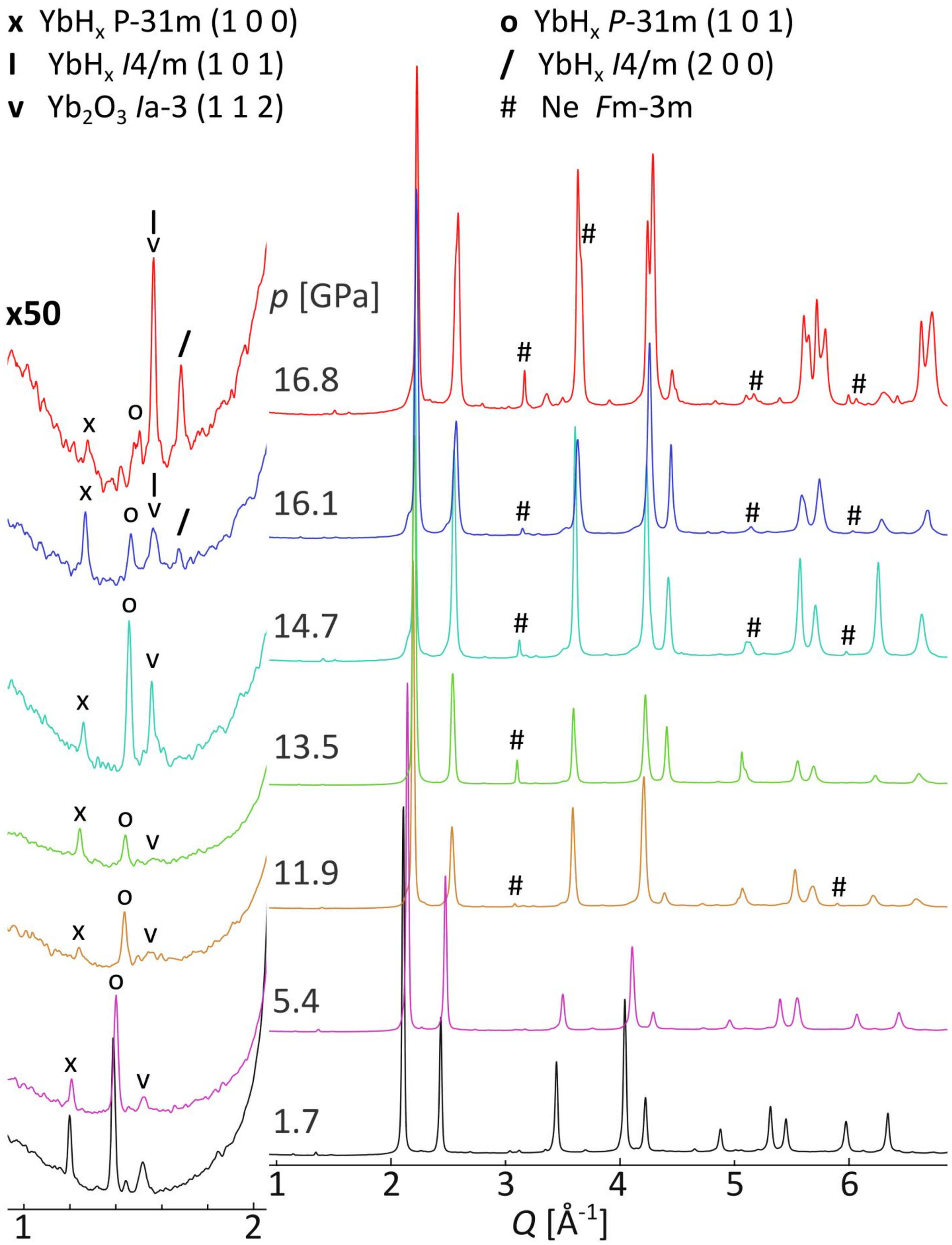

Figure S15. The integrated diffraction data for Yb_3H_8 compressed in Ne (right) with the expanded low-Q region (left). λ = 0.3344 Å. The most visible reflections of the YbH_{2+x} and Yb_2O_3 phases were marked on the low-Q region.

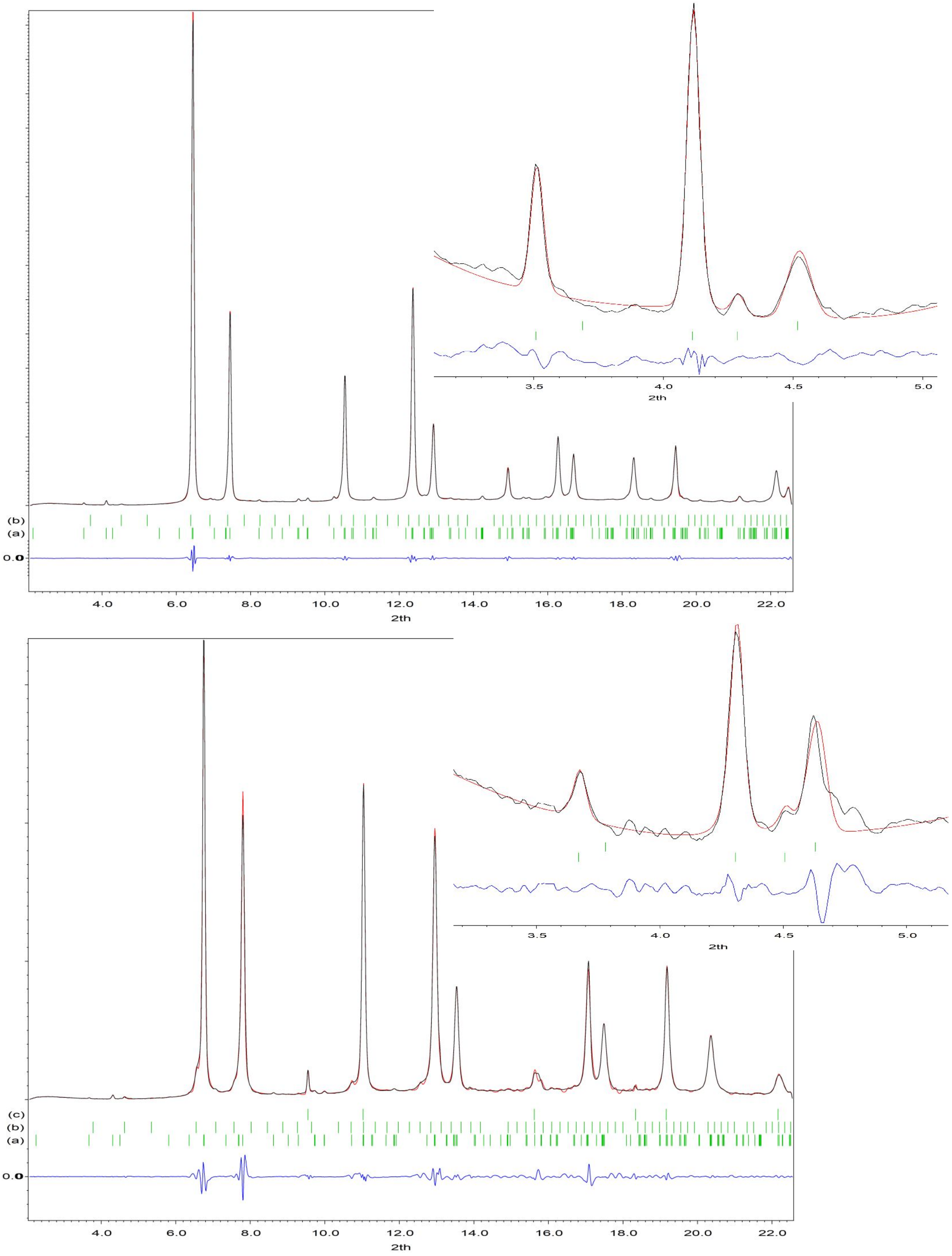

*Figure S16. The diffraction data for Yb_3H_8 compressed in Ne. **p** = 1.7 GPa (top) and 14.7 GPa (bottom), (a) – YbH_{2+x} P-31m, (b) – Yb_2O_3 Ia-3, (c) – Ne Fm-3m. LeBail fit has been marked with a red line, while the positions of Bragg reflections and the difference curve have been plotted at the bottom. The low-angle area is shown as an inset. λ = 0.3344 Å.*

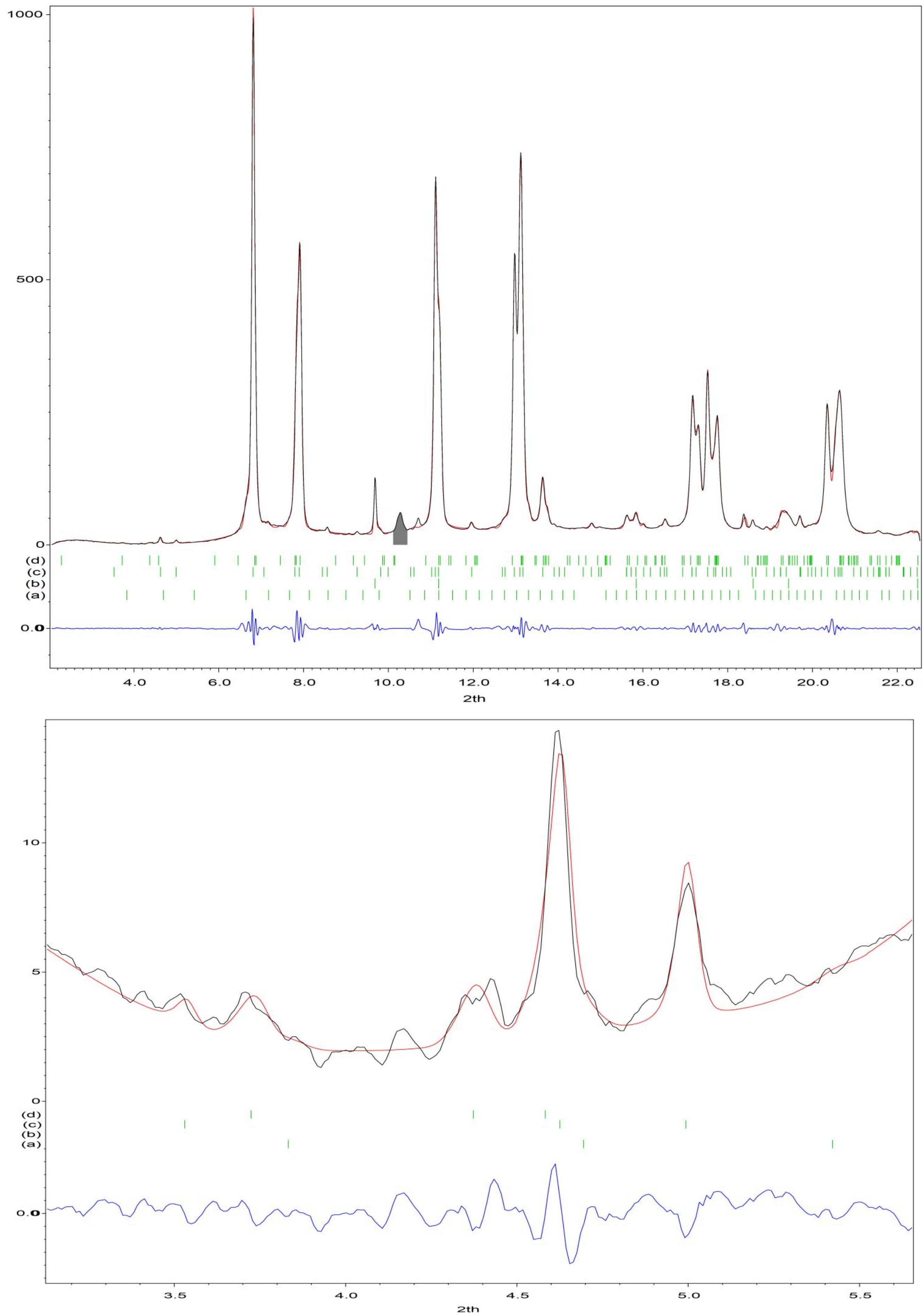

Figure S17. The diffraction data for Yb_3H_8 compressed in Ne. ***p*** *= 16.8 GPa, (a) – Yb_2O_3 Ia-3, (b) – Ne Fm-3m, (c) – YbH_{2+x} I4/m, (d) – YbH_{2+x} P-31m. LeBail fit has been marked with a red line, while the positions of Bragg reflections and the difference curve have been plotted at the bottom. The low-angle area is shown at the bottom. λ = 0.3344 Å.*

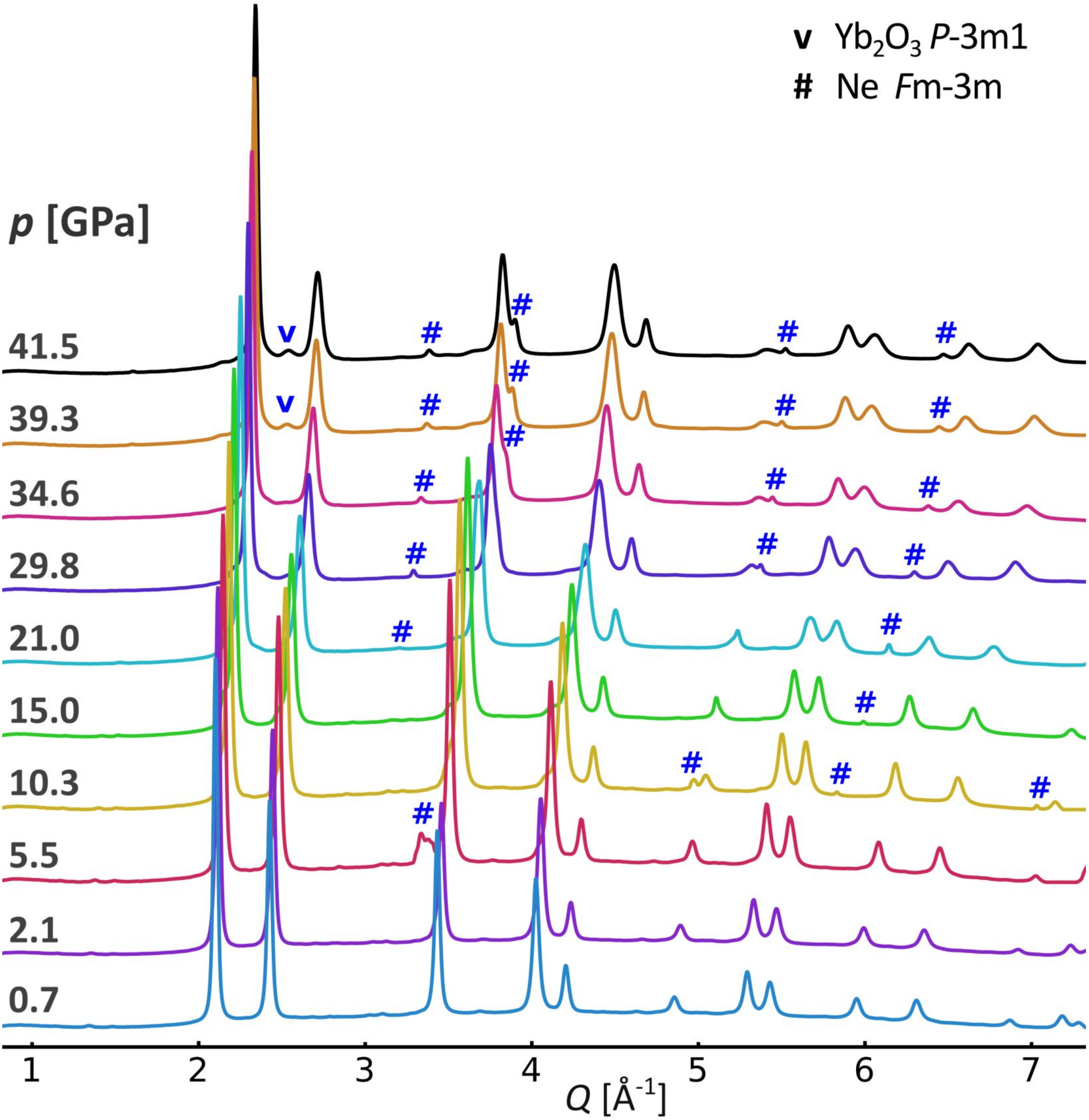

Figure S18. The integrated diffraction data for Yb_3H_8 compressed in Ne. λ = 0.2952 Å. The most visible reflections of the Yb_2O_3 and Ne phases were marked.

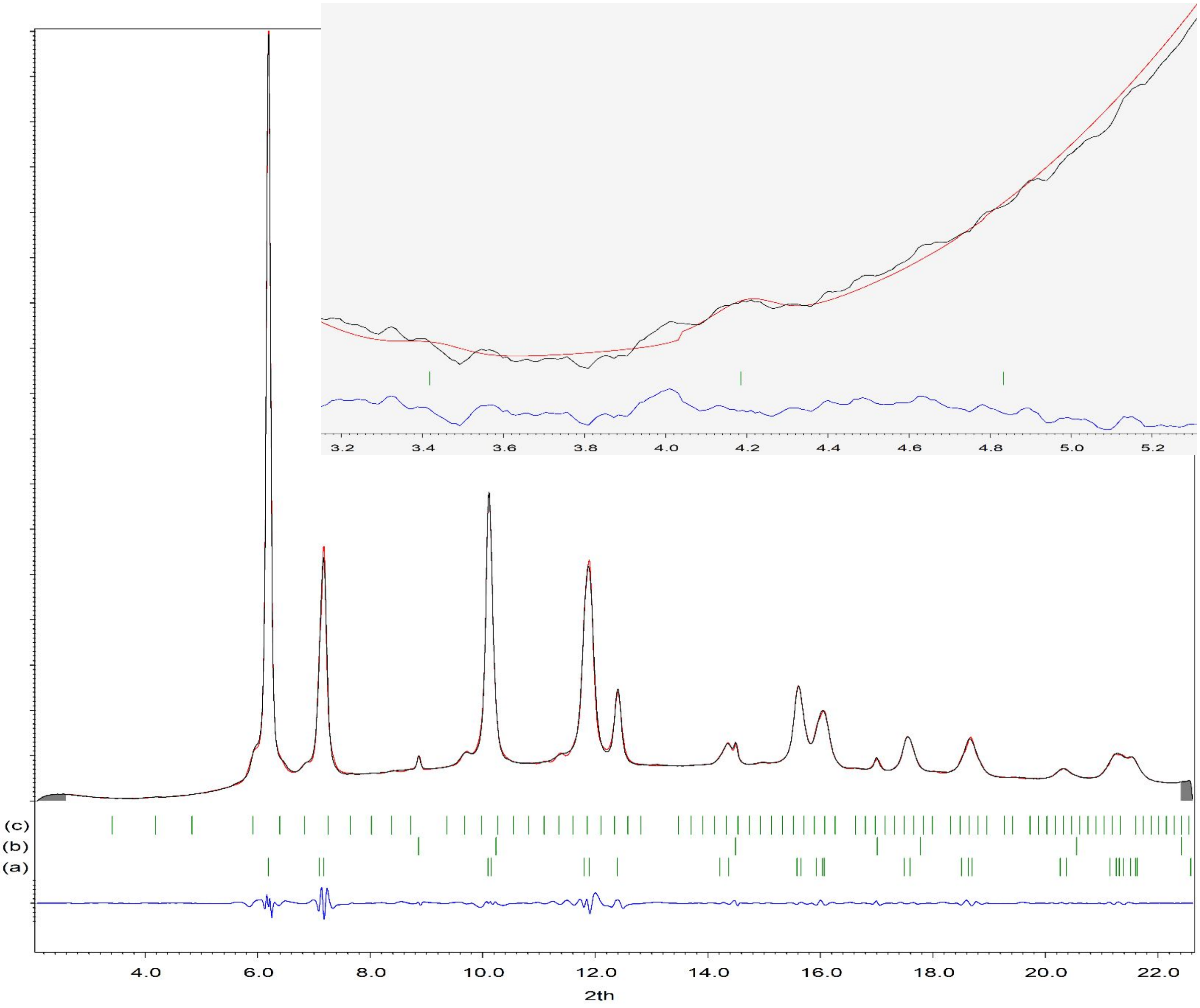

Figure S19. The diffraction data for Yb_3H_8 compressed in Ne. ***p*** *= 28.9 GPa, (a) – YbH_{2+x} I4/mmm, (b) – Ne Fm-3m, (c) – Yb_2O_3 Ia-3. LeBail fit has been marked with a red line, while the positions of Bragg reflections and the difference curve have been plotted at the bottom. The low-angle area is shown as an inset (ca. 80x magnification of the ordinate). λ = 0.2952 Å.*

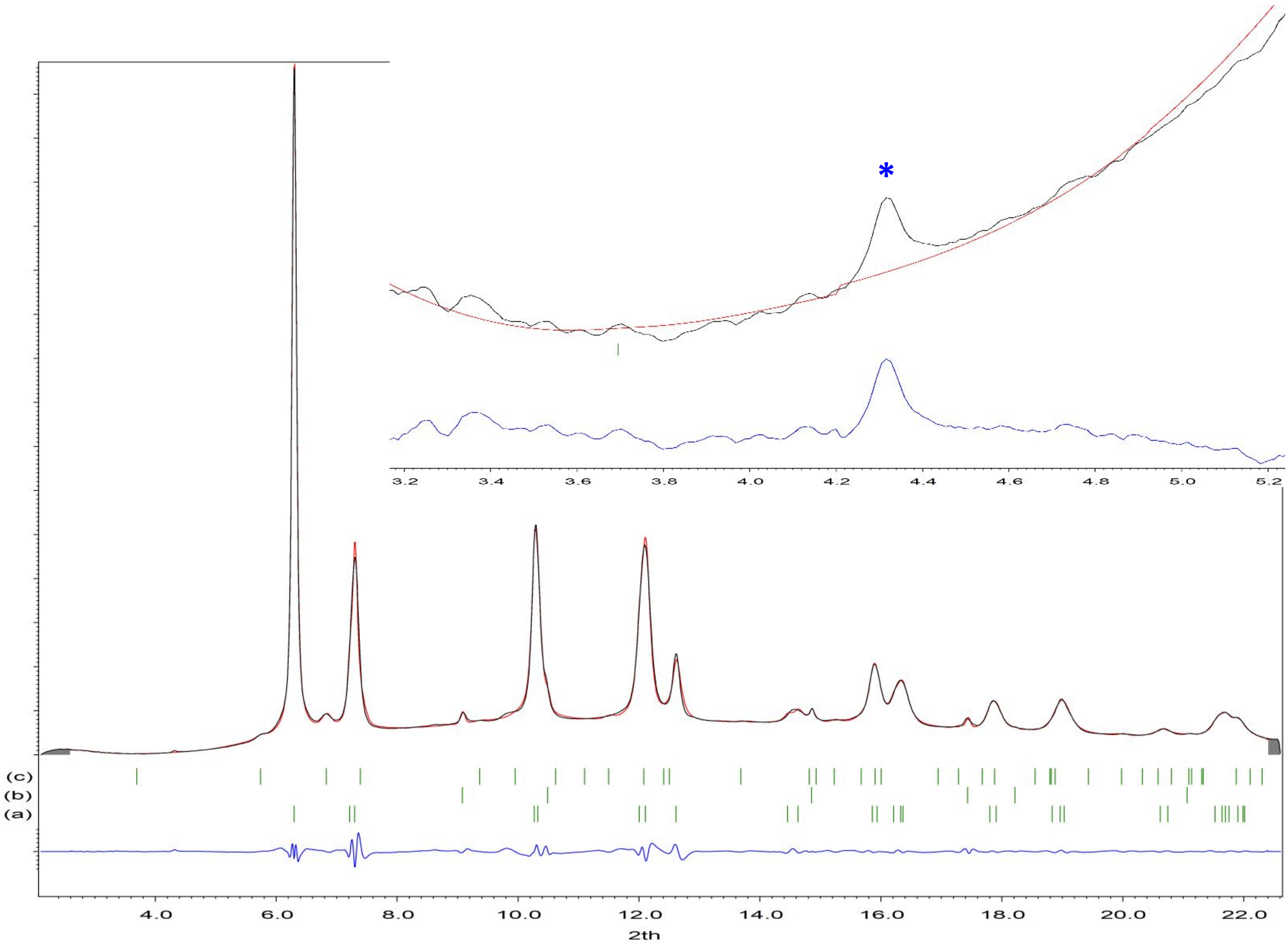

Figure S20. The diffraction data for Yb_3H_8 compressed in Ne. ***p*** *= 39.3 GPa, (a) – YbH_{2+x} I4/mmm, (b) – Ne Fm-3m, (c) – Yb_2O_3 P-3m1; * – unidentified signal. LeBail fit has been marked with a red line, while the positions of Bragg reflections and the difference curve have been plotted at the bottom. The low-angle area is shown as an inset (ca. 80x magnification of the ordinate). λ = 0.2952 Å.*

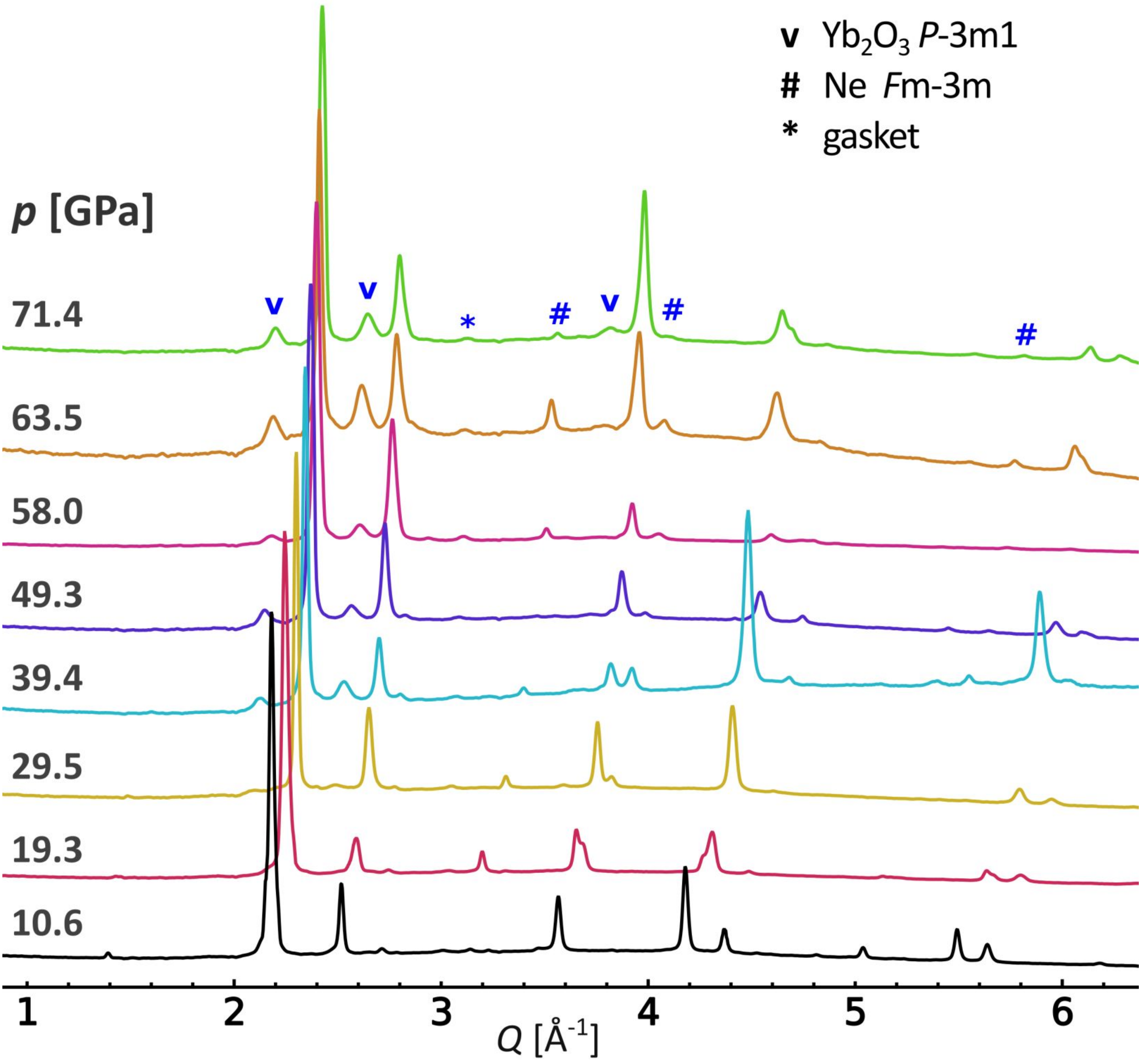

Figure S21. The integrated diffraction data for Yb_3H_8 compressed in Ne. λ = 0.3445 Å. The most visible reflections of the Yb_2O_3 and Ne phases were marked.

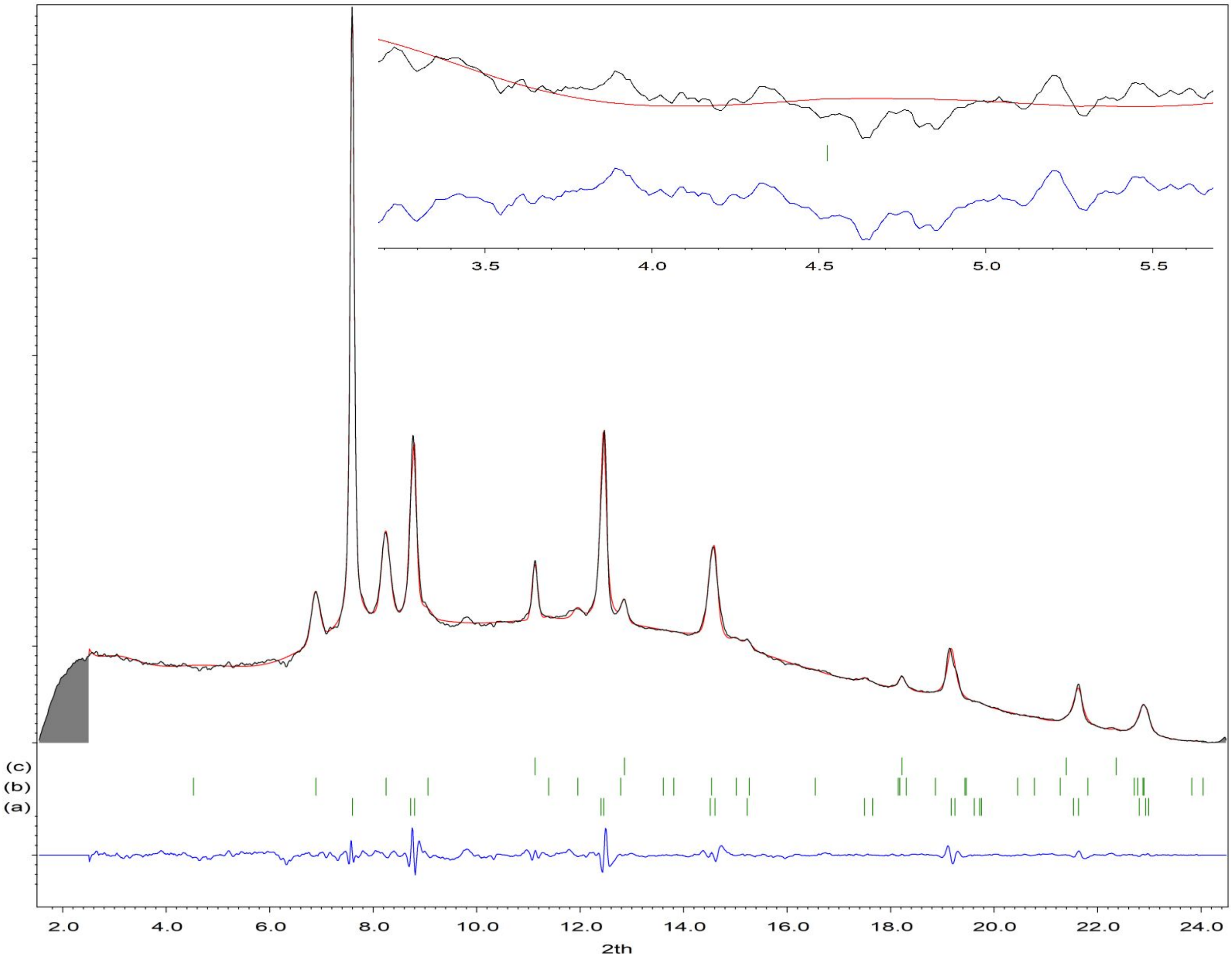

Figure S22. The diffraction data for Yb_3H_8 compressed in Ne. ***p*** *= 63.5 GPa, (a) – YbH_{2+x} I4/mmm, (b) – Yb_2O_3 P-3m1, (c)– Ne Fm-3m. LeBail fit has been marked with a red line, while the positions of Bragg reflections and the difference curve have been plotted at the bottom. The low-angle area is shown as an inset (ca. 80x magnification of the ordinate). λ = 0.3445 Å.*

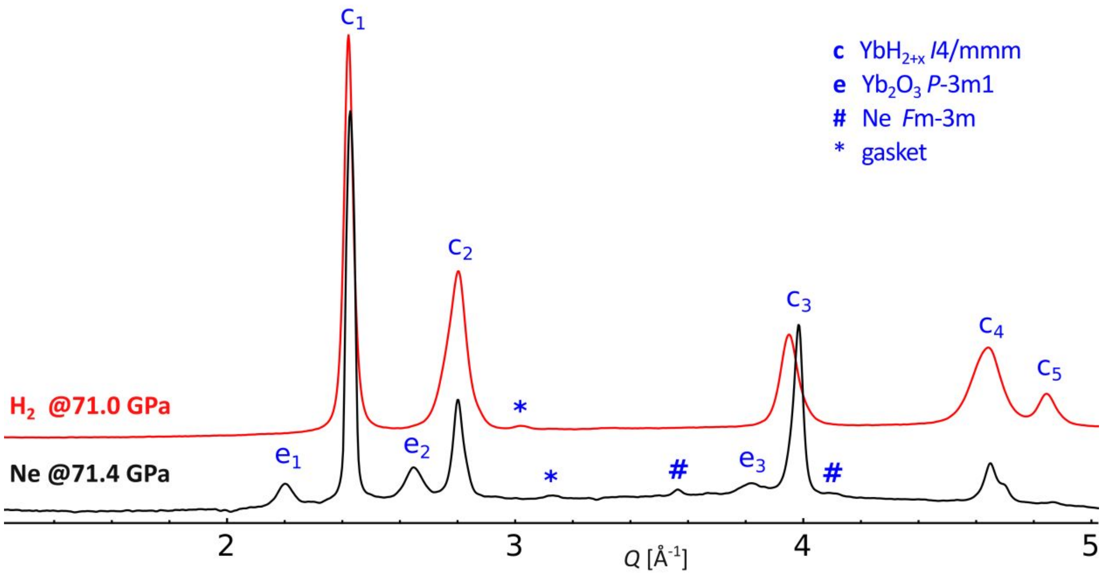

Figure S23. A comparison of the integrated diffraction data for Yb_3H_8 compressed to ca. 71 GPa in Ne and in H_2. λ = 0.3445 Å (Ne) and 0.4066 Å (H_2). The most visible reflections of the YbH_{2+x} and Yb_2O_3 phases were marked: c – YbH_{2+x} I4/mmm: 1-(1 0 1), 2-(0 0 2) and (1 1 0), 3-(1 1 2) and (2 0 0), 4-(1 0 3) and (2 1 1), 5-(2 0 2); e – Yb_2O_3 P-3m1: 1-(1 0 0), 2-(1 0 1), 3-(2 -1 0).

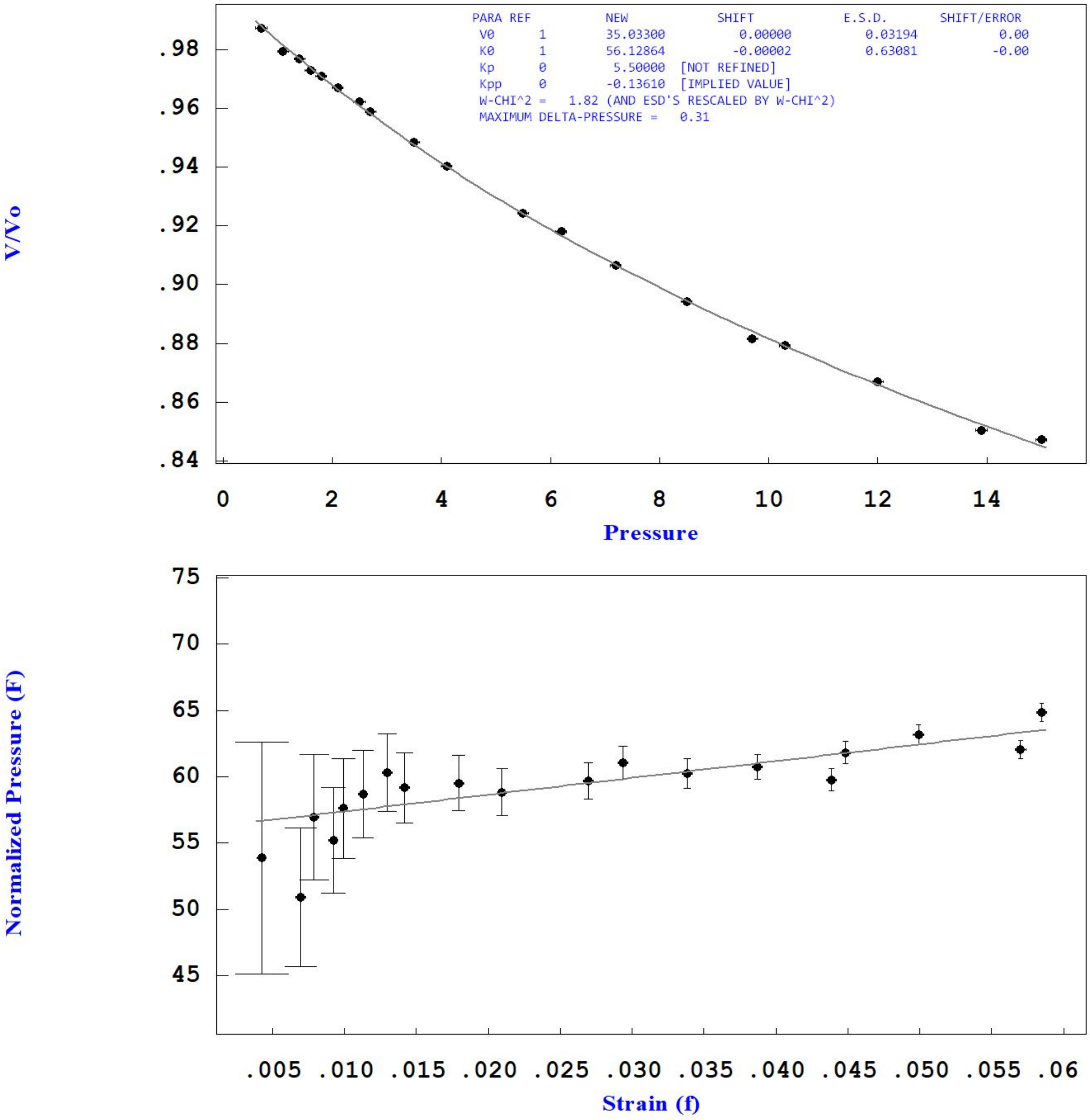

Figure S24. The fit of the volume per Yb atom to the third-order Birch-Murnaghan equation of state for P–31m phase of Yb_3H_8 compressed in Ne PTM (top) and the F–f plot (bottom). F – normalized pressure [GPa], f – Eulerian strain; $F = P/3f(1+2f)^{5/2}$, $f = [(V_0/V)^{2/3} – 1]/2$, $F = K_0 + [3K_0(K' – 4)/2]f$. The experimental data reveal the positive slope of the F–f plot, indicating K' > 4.[1] *Run (g).*

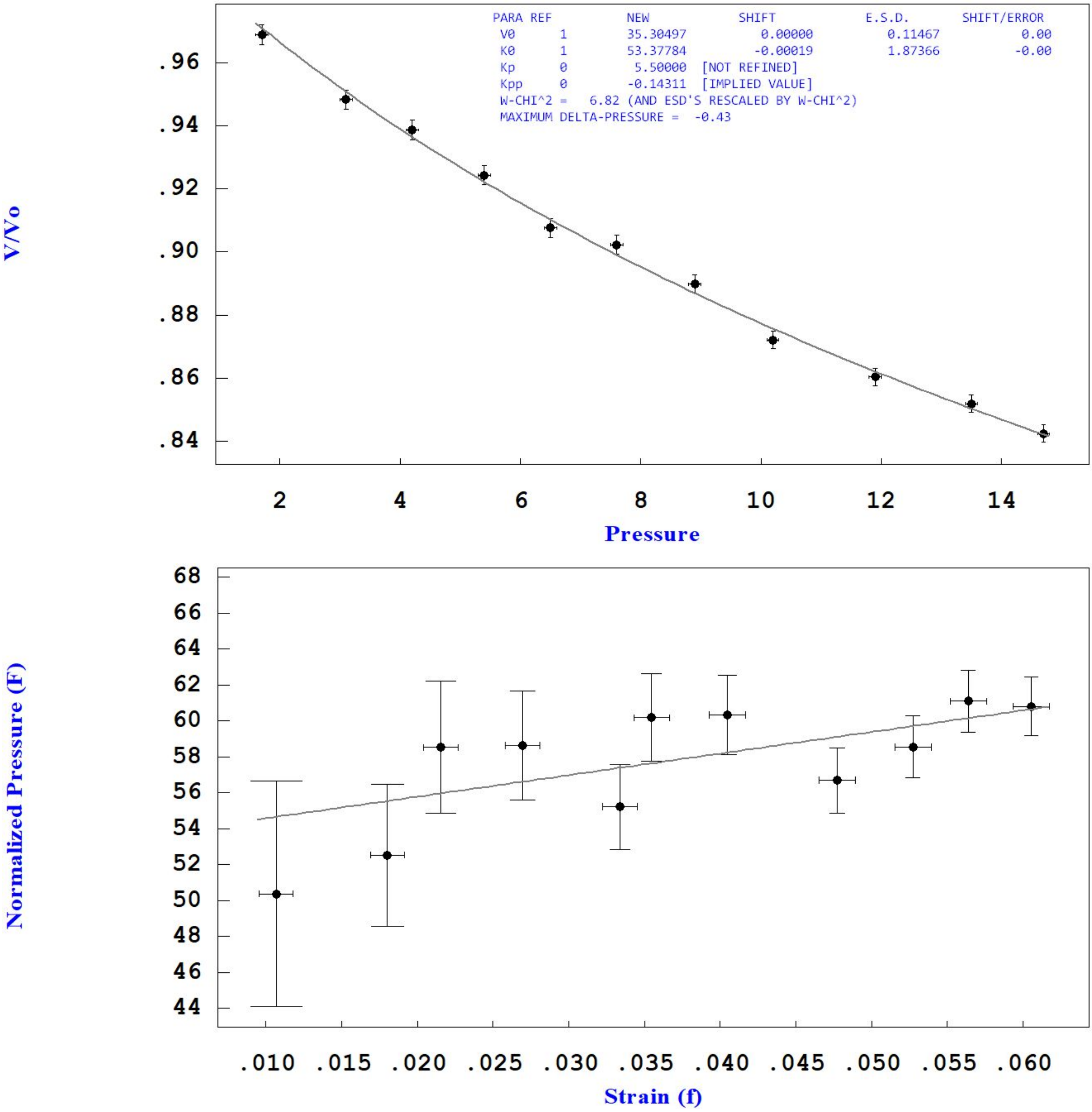

Figure S25. The fit of the volume per Yb atom to the third-order Birch-Murnaghan equation of state for P–31m phase of Yb_3H_8 *compressed in Ne PTM (top) and the F–f plot (bottom). F – normalized pressure [GPa], f – Eulerian strain. Alternative run (h).*

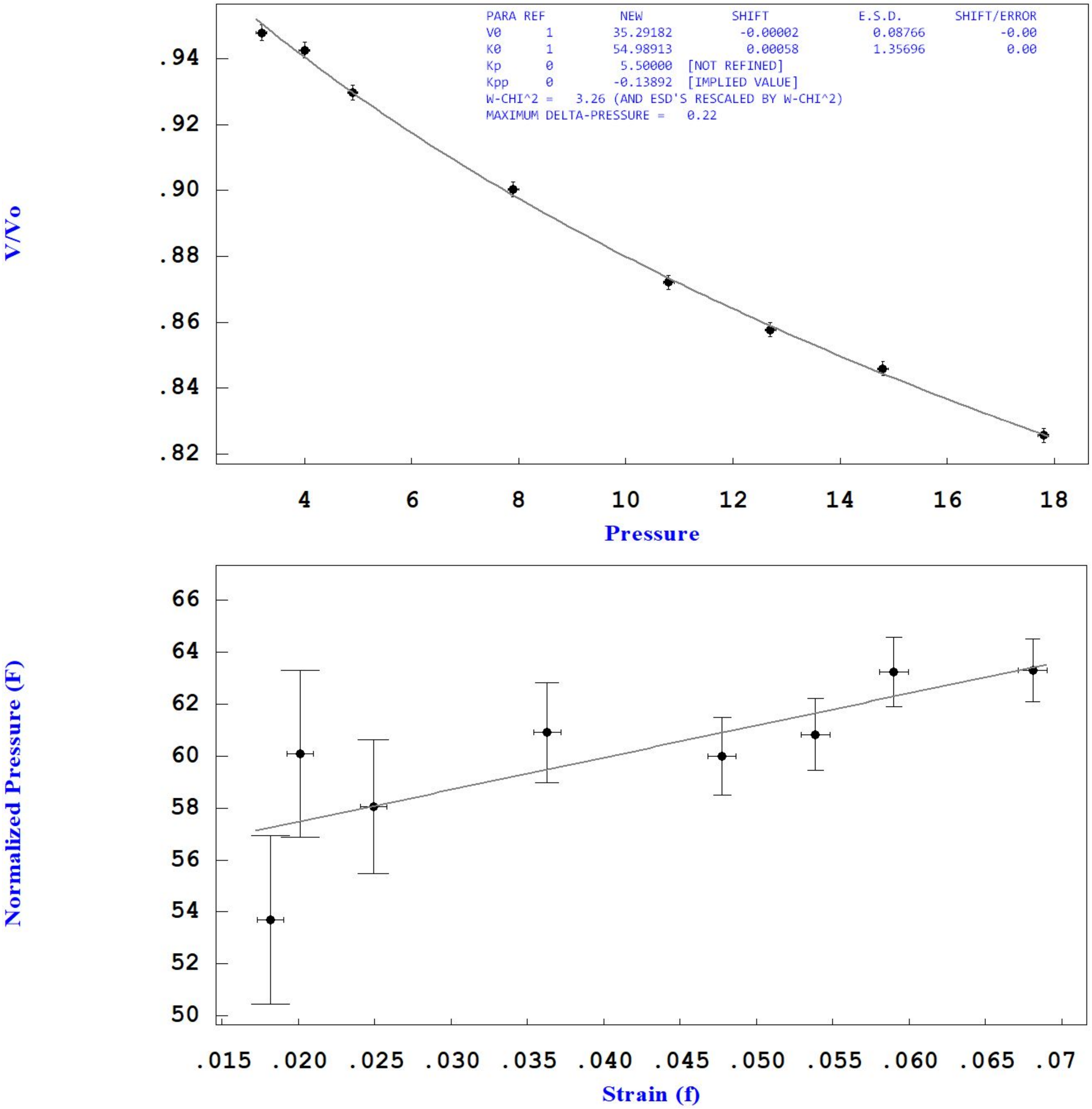

Figure S26. The fit of the volume per Yb atom to the third-order Birch-Murnaghan equation of state for P–31m phase of Yb_3H_8 compressed in H_2 PTM (top) and the F–f plot (bottom). F – normalized pressure [GPa], f – Eulerian strain. Run (d).

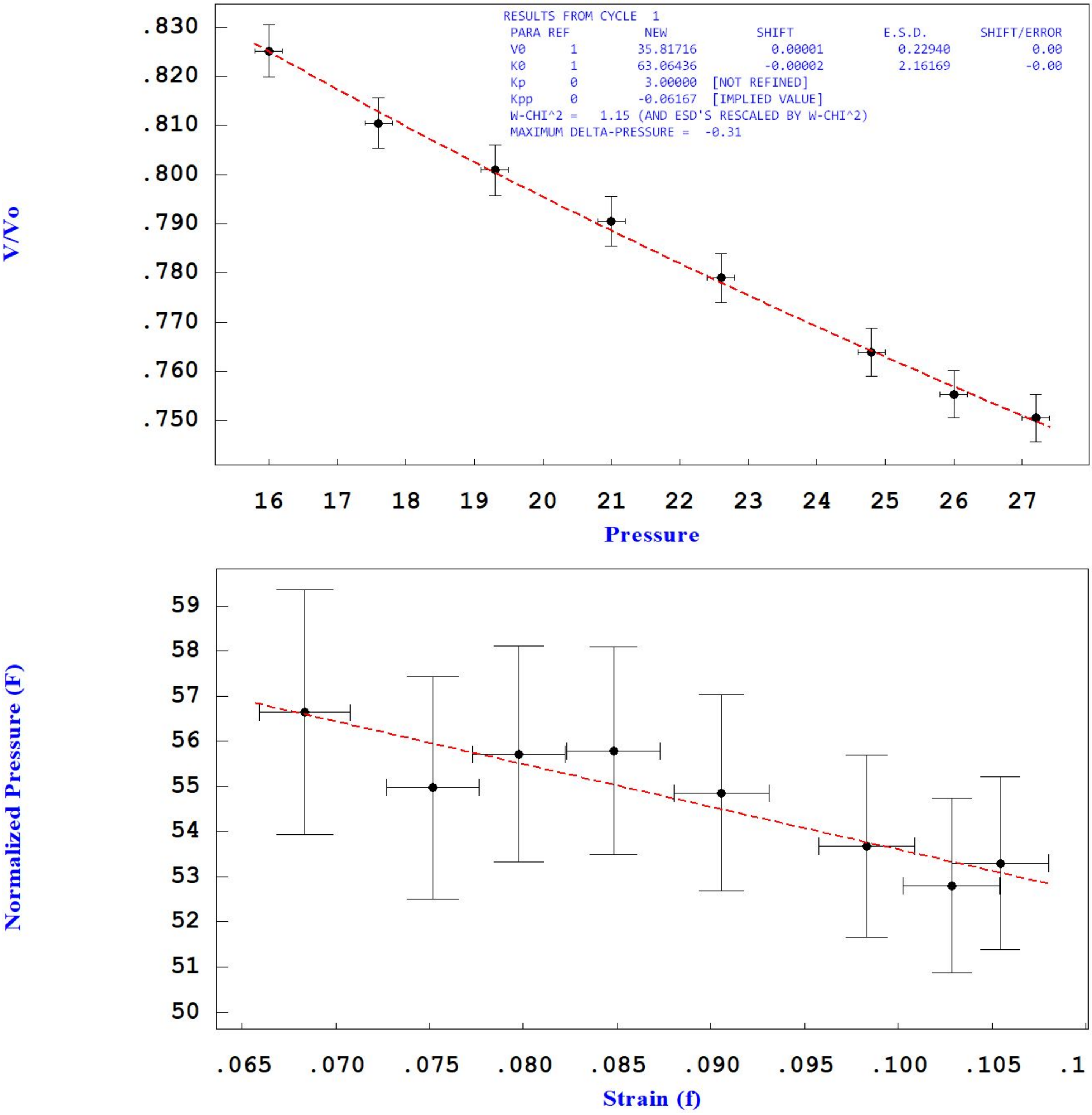

Figure S27. The fit of the volume per Yb atom to the third-order Birch-Murnaghan equation of state for I4/m phase of Yb_3H_8 *compressed in Ne PTM (top) and the F–f plot (bottom). F – normalized pressure [GPa], f – Eulerian strain. Run (g).*

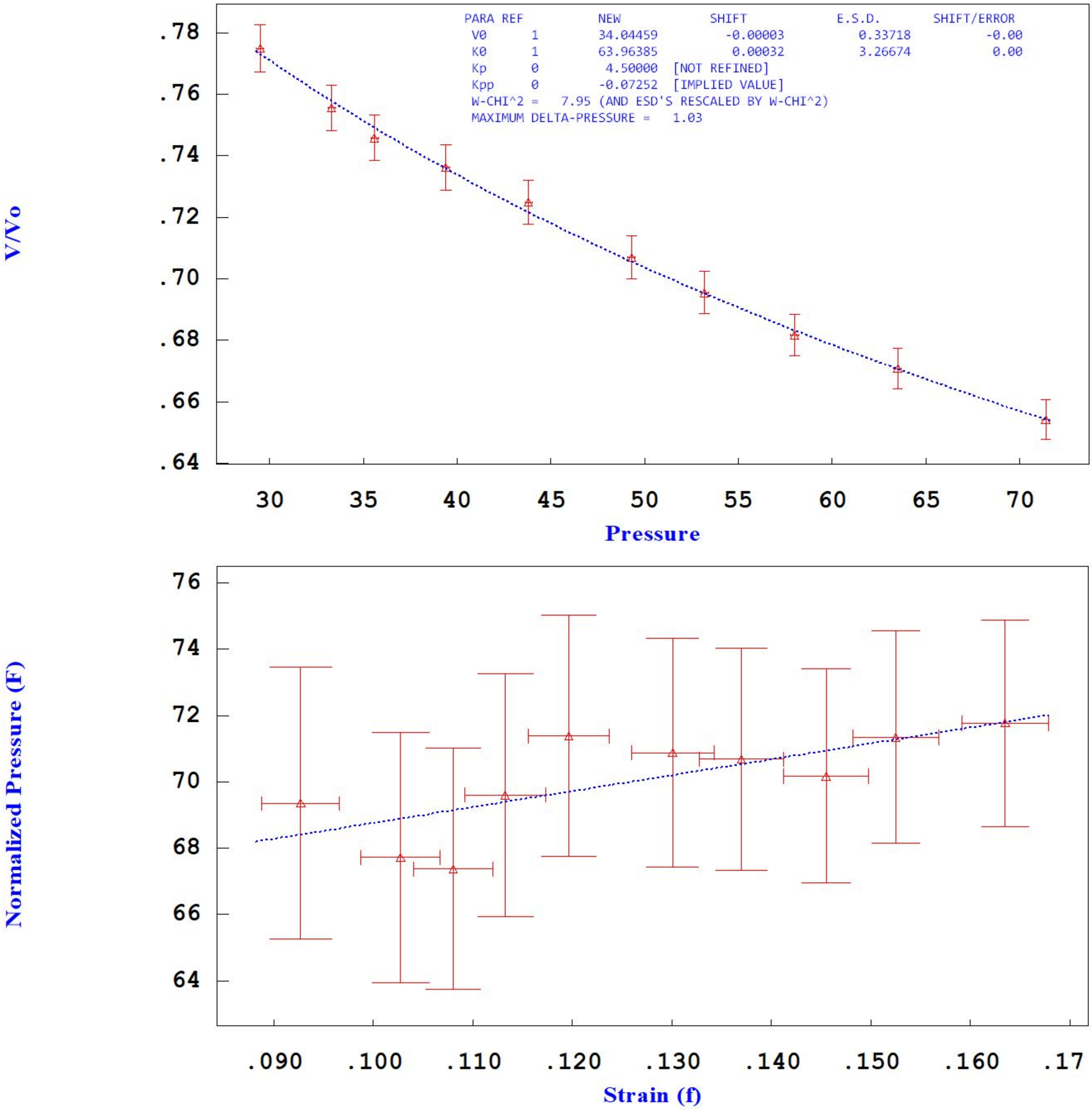

Figure S28. The fit of the volume per Yb atom to the third-order Birch-Murnaghan equation of state for I4/mmm phase of Yb_3H_8 compressed in Ne PTM (top) and the F–f plot (bottom). F – normalized pressure [GPa], f – Eulerian strain. run (i).

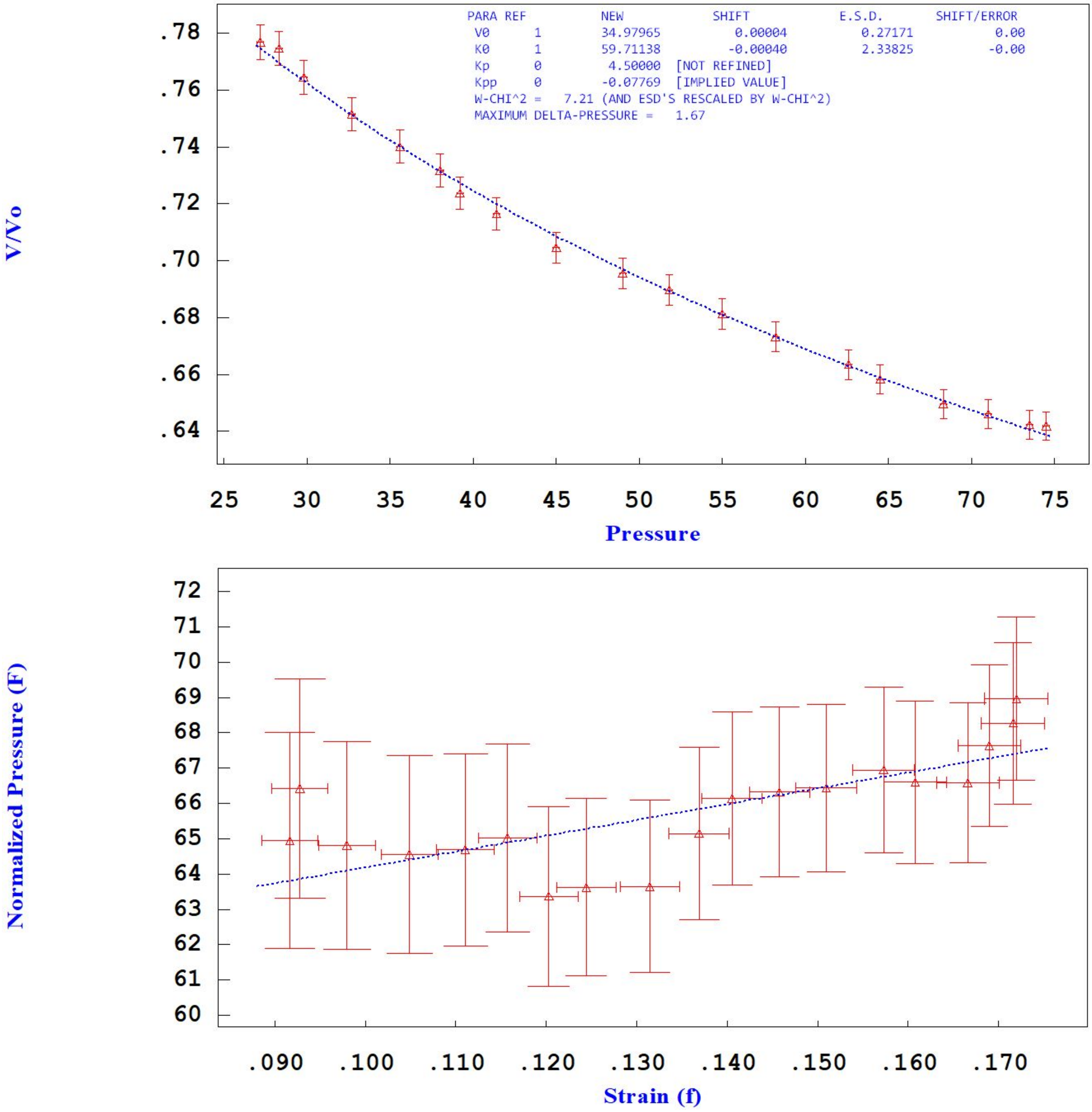

Figure S29. The fit of the volume per Yb atom to the third-order Birch-Murnaghan equation of state for I4/mmm phase of Yb_3H_8 *compressed in* H_2 *PTM (top) and the F–f plot (bottom). F – normalized pressure [GPa], f – Eulerian strain. run (d).*

Table S2. Summary of the EoS parameters reported for some of the lanthanide hydrides relevant for this study. The estimated standard deviations (E.S.D.) are given in parentheses. EoS type: B-M – Birch-Murnaghan, M – Murnaghan.

phase	V_0 per metal atom [Å^3]	B_0 [GPa]	B_0'	EoS type	notes	ref.
**SmH_3** *P*6$_3$/mmc	41.68(33)	70(4)	4	M	silicone oil PTM	[2]
**SmH_3** *F*m-3m	38.69(33)	80(4)	4	M	silicone oil PTM	[2]
**EuH_2** *P*nma	42.6	39.9(5)	4	B-M	He PTM	[3]
**EuH_2** *P*6$_3$/mmc	39.8(1)	44.9(9)	4	B-M	He PTM	[3]
**EuH_{2+x}** *I*4/mmm	38.8(2)	69(2)	4	B-M	H_2 PTM; *I*4/m phase shows similar compressibility	[3]
**Eu_8H_{46}** *P*m-3n	26.3(1)	471(70)	4	B-M	DFT – close to exp. data; V_0 at 100 GPa	[4]
**EuH_9** *P*6$_3$/mmc	31.9(1)	594(70)	4	B-M	DFT – close to exp. data; V_0 at 100 GPa	[4]
**EuH_9** *F*-43m	31.3(1)	699(27)	4	B-M	DFT – close to exp. data; V_0 at 100 GPa	[4]
**TbH_3** *P*6$_3$/mmc	39.69	81	4	M	no PTM	[5]
**TbH_3** *F*m-3m	38.03	96	4	M	no PTM	[5]
**DyH_3** *P*6$_3$/mmc	38.53	82	5.1	M	silicone oil PTM	[6]
**DyH_3** *F*m-3m	35.54	119	1.9	M	silicone oil PTM	[6]
**DyH_3** *F*m-3m	35.4(3)	85(3)	4	B-M	H_2 PTM	[7]
**HoH_3** *P*6$_3$/mmc	37.69	87(3)	4	M	silicone oil PTM	[8]
**HoH_3** *F*m-3m	36.20	90(2)	4	M	silicone oil PTM	[8]
**ErH_3** *P*6$_3$/mmc	37.03	77(4)	4	M	silicone oil PTM	[2]
**ErH_3** *F*m-3m	35.70	81(3)	4	M	silicone oil PTM	[2]
**TmH_3** *P*-3c1	35.69	84.94	3.22	B-M	DFT	[9]
**TmH_3** *F*m-3m	32.06	103.36	3.86	B-M	DFT	[9]
**YbH_2** *P*nma	35.63(7)	40.2(22)	4.75(45)	M	4:1 MeOH:EtOH PTM	[10]
**YbH_2** *P*6$_3$/mmc	30.4(1)	138(3)	0	M	4:1 MeOH:EtOH PTM	[10]

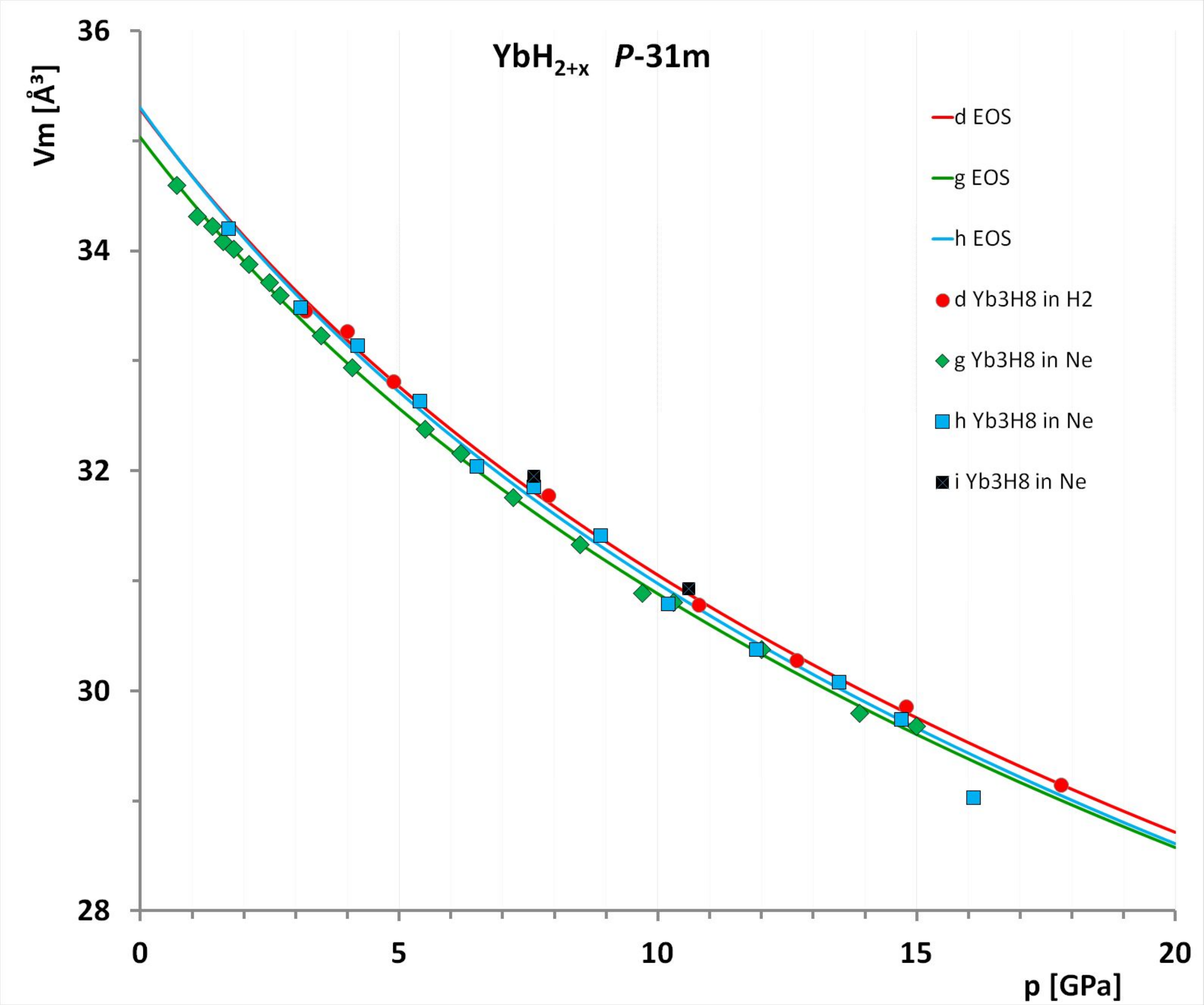

Figure S30. P–31m phase of Yb_3H_8 compressed in H_2 and Ne PTM. Comparison of the experimental data and EOS fitted.

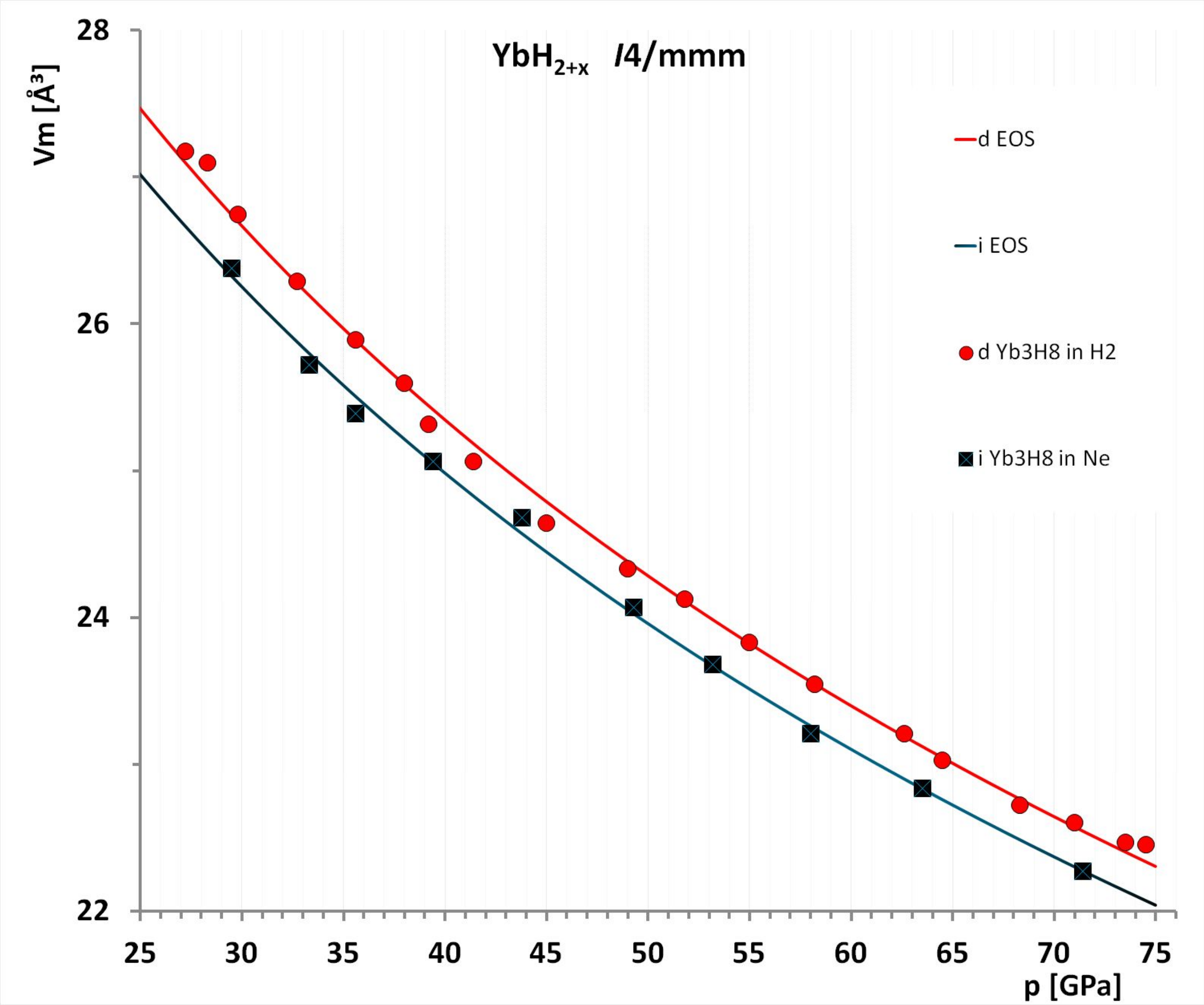

Figure S31. I4/mmm phase of Yb_3H_8 compressed in H_2 and Ne PTM. Comparison of the experimental data and EOS fitted.

Table S3. Refined lattice parameters and their estimated standard deviations (e.s.d.) for Yb_3H_8 compressed in H_2 PTM. Run (d).

**p [GPa]**	**YbH_{2+x} phase**	**a [Å]**	**a (e.s.d.)**	**c [Å]**	**c (e.s.d)**	**V/Z [Å^3]**	**c/a**	**cwRp [%]**
3.2	*P*-31m	6.2625	9	8.8642	16	33.452	1.415	5.57
4.0		6.2491	4	8.8526	12	33.265	1.417	4.62
4.9		6.21623	13	8.8237	5	32.809	1.419	3.34
7.9		6.1535	4	8.7196	10	31.771	1.417	3.27
10.8		6.0898	5	8.6246	9	30.777	1.416	3.61
12.7		6.0497	4	8.5950	16	30.269	1.421	3.75
14.8		6.0441	5	8.4926	4	29.853	1.405	4.00
17.8		5.9762	8	8.4788	18	29.139	1.419	3.86
14.8	*I*4/m	7.7780	3	4.8923	3	29.597	0.629	4.00
17.8		7.6724	3	4.9259	3	28.997	0.642	3.86
20.6		7.6386	2	4.8978	3	28.578	0.641	5.65
21.7		7.6180	4	4.8849	5	28.349	0.641	7.52
24.0		7.5837	4	4.8612	5	27.958	0.641	6.83
27.2	*I*4/mmm	3.3679	3	4.7916	7	27.175	1.423	6.81
28.3		3.3613	4	4.7966	8	27.097	1.427	7.02
29.8		3.3470	5	4.7748	7	26.745	1.427	6.50
32.7		3.3270	9	4.7498	13	26.288	1.428	8.59
35.6		3.3100	13	4.726	2	25.889	1.428	11.18
38.0		3.2974	7	4.7082	9	25.596	1.428	7.26
39.2		3.2837	8	4.6955	11	25.315	1.430	7.72
41.4		3.2735	9	4.6778	13	25.063	1.429	7.76
45.0		3.2545	11	4.6537	18	24.645	1.430	8.42
49.0		3.2393	4	4.6375	9	24.331	1.432	8.38
51.8		3.2296	13	4.6258	17	24.124	1.432	8.66
55.0		3.2162	11	4.6075	14	23.830	1.433	7.34
58.2		3.2027	13	4.5912	19	23.547	1.434	8.51
62.6		3.1869	14	4.570	2	23.207	1.434	9.10
64.5		3.1778	13	4.560	2	23.024	1.435	8.82
68.3		3.1642	16	4.5392	18	22.724	1.435	6.86
71.0		3.1575	4	4.5341	10	22.602	1.436	8.22
73.5		3.1510	4	4.5261	10	22.469	1.436	7.67
74.5		3.1506	10	4.5236	17	22.451	1.436	8.31

Table S4. Refined lattice parameters and their estimated standard deviations (e.s.d.) for Yb_3H_8 compressed in Ne PTM. Run (g).

**p [GPa]**	**YbH_{2+x} phase**	**a [Å]**	**a (e.s.d.)**	**c [Å]**	**c (e.s.d)**	**V/Z [Å³]**	**c/a**	**cwRp [%]**
0.7	*P*-31m	6.3317	6	8.967	3	34.592	1.416	5.31
1.1		6.3189	8	8.9311	18	34.314	1.413	5.47
1.4		6.3144	5	8.9195	12	34.221	1.413	4.78
1.6		6.3062	9	8.9072	12	34.085	1.412	4.82
1.8		6.3024	9	8.8999	12	34.016	1.412	4.97
2.1		6.2944	9	8.8869	10	33.880	1.412	4.81
2.5		6.2836	7	8.8737	11	33.714	1.412	4.43
2.7		6.2704	4	8.8793	16	33.594	1.416	4.78
3.5		6.2485	8	8.8444	17	33.228	1.415	5.31
4.1		6.2297	5	8.8207	16	32.940	1.416	5.40
5.5		6.1930	5	8.7739	15	32.380	1.417	5.89
6.2		6.1862	7	8.7334	16	32.160	1.412	5.43
7.2		6.1605	9	8.696	2	31.757	1.412	6.06
8.5		6.1270	7	8.6720	18	31.326	1.415	6.23
9.7		6.0967	6	8.6347	17	30.883	1.416	6.26
10.3		6.0908	9	8.6283	18	30.801	1.417	6.44
12		6.0617	7	8.5905	20	30.374	1.417	6.73
13.9		6.0427	3	8.4800	14	29.795	1.403	6.30
15		6.0333	9	8.4730	13	29.678	1.404	6.47
16.0	*I*4/m	7.7369	5	4.9373	7	29.554	0.638	4.61
17.6		7.6878	5	4.9117	7	29.029	0.639	5.82
19.3		7.6529	4	4.8981	5	28.687	0.640	4.58
21		7.6211	3	4.8752	5	28.316	0.640	3.41
22.6		7.5853	3	4.8494	6	27.902	0.639	3.79
24.8		7.5378	7	4.8156	7	27.361	0.639	4.36
26		7.5088	4	4.7985	5	27.055	0.639	4.27
27.2		7.4926	4	4.7883	5	26.881	0.639	4.71
28.9	*I*4/mmm	3.3381	4	4.7733	7	26.594	1.430	5.34
29.8		3.332	4	4.7637	6	26.444	1.430	5.12
30.6		3.324	3	4.7535	5	26.261	1.430	5.73
32.3		3.3154	2	4.7425	6	26.064	1.430	5.48
33.3		3.3079	4	4.7316	6	25.887	1.430	5.15
34.6		3.3036	7	4.7252	10	25.785	1.430	6.95
35.7		3.296	8	4.7148	14	25.610	1.430	7.35
37.9		3.2882	7	4.7042	13	25.432	1.431	7.09
39.3		3.2794	7	4.6923	13	25.232	1.431	7.21
41.5		3.2681	7	4.6783	16	24.983	1.432	7.92

Table S5. Refined lattice parameters and their estimated standard deviations (e.s.d.) for Yb_3H_8 compressed in Ne PTM. Run (i).

**p [GPa]**	**YbH_{2+x} phase**	**a [Å]**	**a (e.s.d.)**	**c [Å]**	**c (e.s.d)**	**V/Z [Å^3]**	**c/a**	**cwRp [%]**
7.6	*P*-31m	6.15735	9	8.7581	3	31.951	1.422	4.32
10.6		6.0946	2	8.6523	7	30.925	1.420	6.83
17.1	*I*4/m	7.7117	3	4.8952	5	29.112	0.635	5.56
19.3		7.6438	7	4.8827	8	28.528	0.639	8.70
23.9		7.5509	3	4.8080	4	27.413	0.637	6.50
29.5	*I*4/mmm	3.3469	2	4.7099	6	26.380	1.407	7.85
33.3		3.3054	2	4.7086	5	25.722	1.425	8.73
35.6		3.2930	5	4.6825	4	25.388	1.422	7.99
39.4		3.2797	5	4.6601	8	25.063	1.421	11.59
43.8		3.2619	6	4.6386	10	24.677	1.422	8.88
49.3		3.2344	4	4.6011	6	24.067	1.423	10.51
53.2		3.2185	2	4.5714	5	23.677	1.420	8.70
58		3.1957	5	4.5453	7	23.209	1.422	8.22
63.5		3.17532	19	4.5299	7	22.837	1.427	10.74
71.4		3.1497	2	4.4900	7	22.272	1.426	12.51

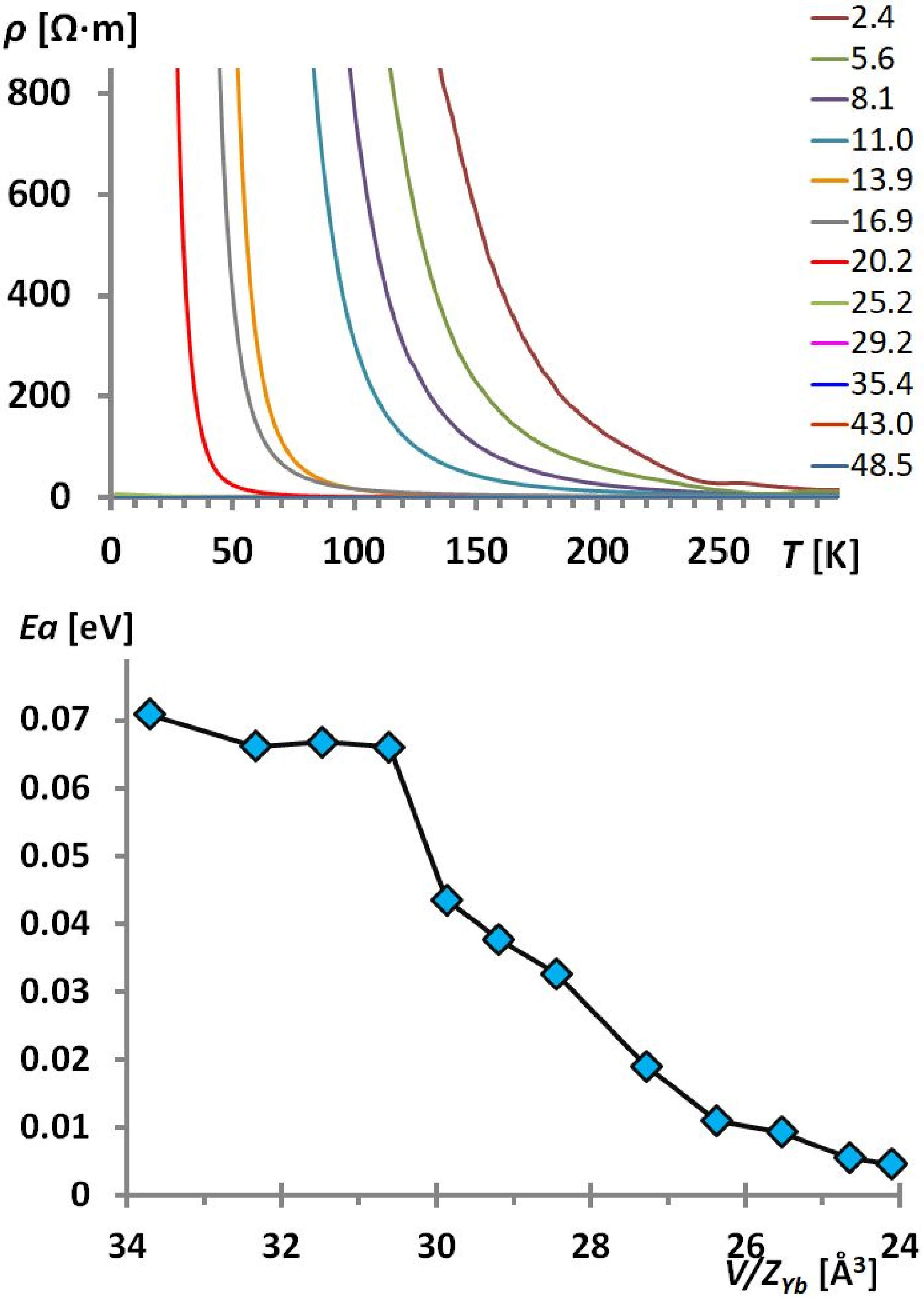

Figure S32. The resistivity vs. T [K] for Yb_3H_8 compressed in NaCl presented in a linear scale indicating semiconductor-like behavior (top). Evolution of the activation energy of the electronic transport in the function of volume per ytterbium atom (bottom).

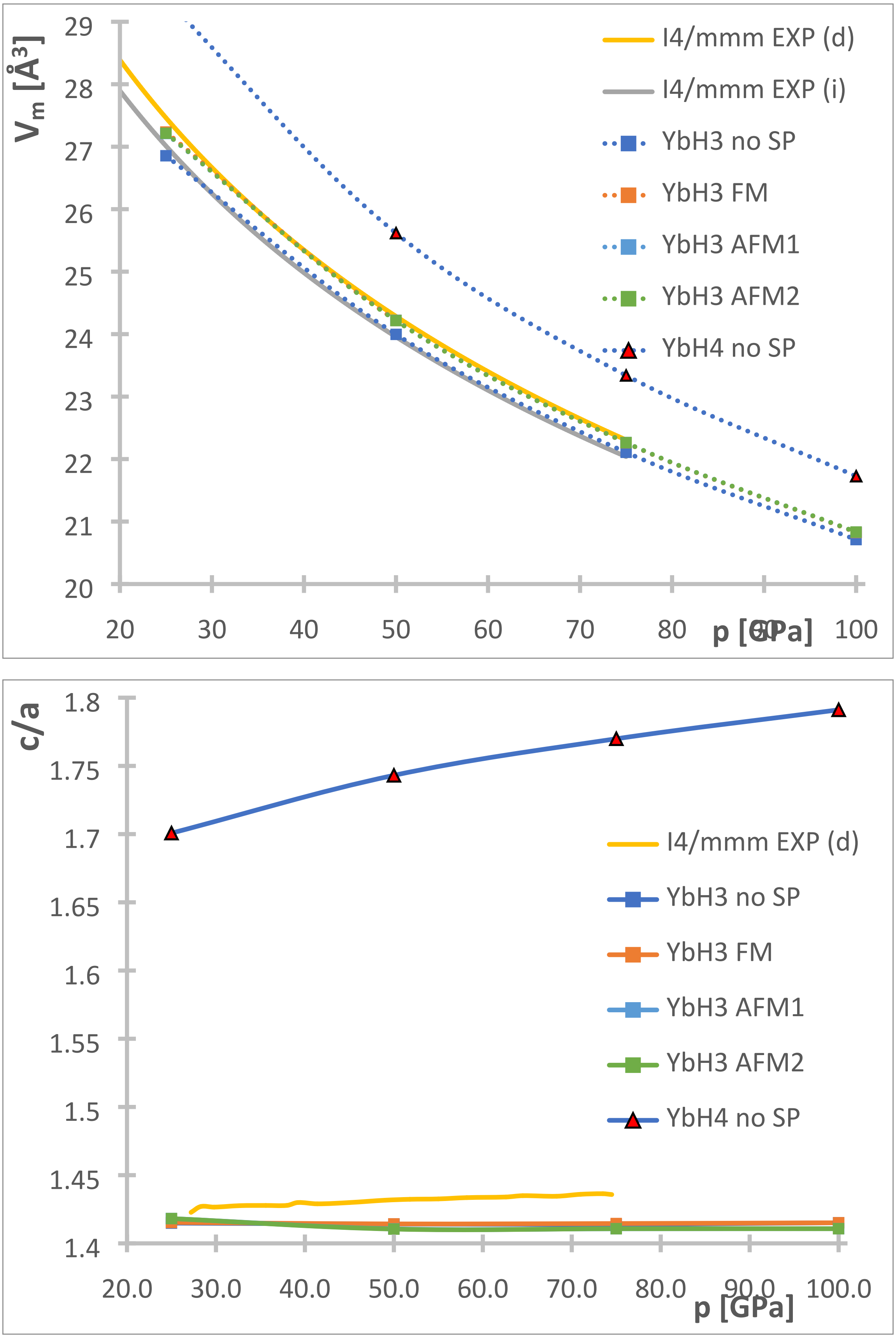

Figure S33. Top – The calculated molar volume of YbH_3 and YbH_4 (both I4/mmm) compared to the experimental results. Bottom – the calculated c/a ratio as compared to the experimental results. EXP run (d) – H_2 PTM, EXP run (i) – Ne PTM.

CIF files: basic parameters, experimental data

Yb_3H_8 *P*-31m

Yb3H8 in Ne, 1.7 GPa (run (h)); LeBail fit (*cf. Fig. S16*)

```
#======================================================================
# CRYSTAL DATA
#----------------------------------------------------------------------
data_ phase_1

_chemical_formula_sum                'H2.67 Yb1'
_cell_length_a                      6.30366(9)
_cell_length_b                      6.30366(9)
_cell_length_c                      8.9455(3)
_cell_angle_alpha                     90
_cell_angle_beta                      90
_cell_angle_gamma                     120
_cell_volume                        307.835(12)
_space_group_name_H-M_alt            'P -3 1 m'
_space_group_IT_number               162

loop_
_space_group_symop_operation_xyz
  'x, y, z'
  '-x, -y, -z'
  '-y, x-y, z'
  'y, -x+y, -z'
  '-x+y, -x, z'
  'x-y, x, -z'
  '-y, -x, -z'
  'y, x, z'
  '-x+y, y, -z'
  'x-y, -y, z'
  'x, x-y, -z'
  '-x, -x+y, z'

loop_
  _atom_site_label
  _atom_site_occupancy
  _atom_site_fract_x
  _atom_site_fract_y
  _atom_site_fract_z
  _atom_site_adp_type
  _atom_site_U_iso_or_equiv
  _atom_site_type_symbol
  Yb1     1.0    0.000000    0.000000    0.000000    Uiso  0.025800 Yb
  Yb2     1.0    0.333333    0.666667    0.000000    Uiso  0.025800 Yb
  Yb3     1.0    0.3484(7)   0.000000    0.3292(13)  Uiso  0.025800 Yb
  H1      1.0    0.000000    0.000000    0.644000    Uiso  0.019000 H
  H2      1.0    0.333333    0.666667    0.219000    Uiso  0.019000 H
  H3      1.0    0.322000    0.000000    0.581200    Uiso  0.019000 H
  H4      1.0    0.356000    0.000000    0.075700    Uiso  0.019000 H
  H5      1.0    0.236400    0.000000    0.831900    Uiso  0.019000 H
```

“YbH_3” *I*4/m

Yb + H_2, sample heated *in situ*, *ca.* 12.5 GPa (run (c)); Rietveld fit (*cf. Fig. S8*)

```
#======================================================================
# CRYSTAL DATA
#----------------------------------------------------------------------
data_ phase_2

_chemical_formula_sum                 'H3 Yb1'
_cell_length_a                    7.8268(3)
_cell_length_b                    7.8268(3)
_cell_length_c                    5.0150(3)
_cell_angle_alpha                   90
_cell_angle_beta                    90
_cell_angle_gamma                    90
_cell_volume                      307.21(2)
_space_group_name_H-M_alt            'I 4/m'
_space_group_IT_number              87

loop_
_space_group_symop_operation_xyz
  'x, y, z'
  '-x, -y, -z'
  '-x, -y, z'
  'x, y, -z'
  '-y, x, z'
  'y, -x, -z'
  'y, -x, z'
  '-y, x, -z'
  'x+1/2, y+1/2, z+1/2'
  '-x+1/2, -y+1/2, -z+1/2'
  '-x+1/2, -y+1/2, z+1/2'
  'x+1/2, y+1/2, -z+1/2'
  '-y+1/2, x+1/2, z+1/2'
  'y+1/2, -x+1/2, -z+1/2'
  'y+1/2, -x+1/2, z+1/2'
  '-y+1/2, x+1/2, -z+1/2'

loop_
  _atom_site_label
  _atom_site_occupancy
  _atom_site_fract_x
  _atom_site_fract_y
  _atom_site_fract_z
  _atom_site_adp_type
  _atom_site_U_iso_or_equiv
  _atom_site_type_symbol
  Yb1    1.0   0.000000   0.000000   0.000000   Uiso  0.010000 Yb
  Yb2    1.0   0.4109(7)  0.2013(13)  0.000000   Uiso  0.010000 Yb
  H1    1.0   0.000000   0.000000   0.500000   Uiso  0.010000 H
  H2    1.0   0.091400   0.307700   0.000000   Uiso  0.010000 H
  H3    1.0   0.202200   0.107800   0.239600   Uiso  0.010000 H
  H4    1.0   0.000000   0.500000   0.250000   Uiso  0.010000 H
```

“YbH_3” *I*4/mmm

Yb3H8 in Ne, 28.9 GPa (run (g)); LeBail fit (*cf. Fig. S19*). The H occupancies may vary.

```
#======================================================================
# CRYSTAL DATA
#----------------------------------------------------------------------
data_ phase_3

_chemical_formula_sum                'H3 Yb1'
_cell_length_a                  3.3381(4)
_cell_length_b                  3.3381(4)
_cell_length_c                  4.7733(7)
_cell_angle_alpha                  90
_cell_angle_beta                   90
_cell_angle_gamma                    90
_cell_volume                    53.190(12)
_space_group_name_H-M_alt            'I 4/m m m'
_space_group_IT_number              139

loop_
_space_group_symop_operation_xyz
  'x, y, z'
  '-x, -y, -z'
  '-x, -y, z'
  'x, y, -z'
  '-y, x, z'
  'y, -x, -z'
  'y, -x, z'
  '-y, x, -z'
  '-x, y, -z'
  'x, -y, z'
  'x, -y, -z'
  '-x, y, z'
  'y, x, -z'
  '-y, -x, z'
  '-y, -x, -z'
  'y, x, z'
  'x+1/2, y+1/2, z+1/2'
  '-x+1/2, -y+1/2, -z+1/2'
  '-x+1/2, -y+1/2, z+1/2'
  'x+1/2, y+1/2, -z+1/2'
  '-y+1/2, x+1/2, z+1/2'
  'y+1/2, -x+1/2, -z+1/2'
  'y+1/2, -x+1/2, z+1/2'
  '-y+1/2, x+1/2, -z+1/2'
  '-x+1/2, y+1/2, -z+1/2'
  'x+1/2, -y+1/2, z+1/2'
  'x+1/2, -y+1/2, -z+1/2'
  '-x+1/2, y+1/2, z+1/2'
  'y+1/2, x+1/2, -z+1/2'
  '-y+1/2, -x+1/2, z+1/2'
  '-y+1/2, -x+1/2, -z+1/2'
  'y+1/2, x+1/2, z+1/2'

loop_
  _atom_site_label
  _atom_site_occupancy
```

```
  _atom_site_fract_x
  _atom_site_fract_y
  _atom_site_fract_z
  _atom_site_adp_type
  _atom_site_U_iso_or_equiv
  _atom_site_type_symbol
  Yb1       1.0    0.000000    0.000000    0.000000    Uiso  0.020000 Yb
  H1        1.0    0.000000    0.000000    0.500000    Uiso  0.020000 H
  H2        1.0    0.000000    0.500000    0.250000    Uiso  0.020000 H
```

Yb3H8 in H_2, 73.5 GPa (run (d)); LeBail fit (*cf. Fig. S14*). The H occupancies may vary.

```
#======================================================================
# CRYSTAL DATA
#----------------------------------------------------------------------
data_ phase_3

_chemical_formula_sum                'H3 Yb1'
_cell_length_a                       3.1510(4)
_cell_length_b                       3.1510(4)
_cell_length_c                       4.5261(10)
_cell_angle_alpha                    90
_cell_angle_beta                     90
_cell_angle_gamma                    90
_cell_volume                         44.938(12)
_space_group_name_H-M_alt            'I 4/m m m'
_space_group_IT_number               139

loop_
_space_group_symop_operation_xyz
  'x, y, z'
  '-x, -y, -z'
  '-x, -y, z'
  'x, y, -z'
  '-y, x, z'
  'y, -x, -z'
  'y, -x, z'
  '-y, x, -z'
  '-x, y, -z'
  'x, -y, z'
  'x, -y, -z'
  '-x, y, z'
  'y, x, -z'
  '-y, -x, z'
  '-y, -x, -z'
  'y, x, z'
  'x+1/2, y+1/2, z+1/2'
  '-x+1/2, -y+1/2, -z+1/2'
  '-x+1/2, -y+1/2, z+1/2'
  'x+1/2, y+1/2, -z+1/2'
  '-y+1/2, x+1/2, z+1/2'
  'y+1/2, -x+1/2, -z+1/2'
```

```
   'y+1/2, -x+1/2, z+1/2'
   '-y+1/2, x+1/2, -z+1/2'
   '-x+1/2, y+1/2, -z+1/2'
   'x+1/2, -y+1/2, z+1/2'
   'x+1/2, -y+1/2, -z+1/2'
   '-x+1/2, y+1/2, z+1/2'
   'y+1/2, x+1/2, -z+1/2'
   '-y+1/2, -x+1/2, z+1/2'
   '-y+1/2, -x+1/2, -z+1/2'
   'y+1/2, x+1/2, z+1/2'

loop_
   _atom_site_label
   _atom_site_occupancy
   _atom_site_fract_x
   _atom_site_fract_y
   _atom_site_fract_z
   _atom_site_adp_type
   _atom_site_U_iso_or_equiv
   _atom_site_type_symbol
   Yb1    1.0   0.000000   0.000000   0.000000   Uiso  0.012700 Yb
   H1     1.0   0.000000   0.000000   0.500000   Uiso  0.038000 H
   H2     1.0   0.000000   0.500000   0.250000   Uiso  0.038000 H
```

Supporting references

(1) Jacobsen, S. D.; Holl, C. M.; Adams, K. A.; Fischer, R. A.; Martin, E. S.; Bina, C. R.; Lin, J. F.; Prakapenka, V. B.; Kubo, A.; Dera, P. Compression of Single-Crystal Magnesium Oxide to 118 GPa and a Ruby Pressure Gauge for Helium Pressure Media. *Am. Mineral.* **2008**, *93* (11–12), 1823–1828. https://doi.org/10.2138/am.2008.2988.

(2) Palasyuk, T.; Tkacz, M. Pressure-Induced Structural Phase Transition in Rare-Earth Trihydrides. Part II. SmH3 and Compressibility Systematics. *Solid State Commun.* **2007**, *141* (5), 302–305. https://doi.org/10.1016/j.ssc.2006.06.045.

(3) Matsuoka, T.; Fujihisa, H.; Hirao, N.; Ohishi, Y.; Mitsui, T.; Masuda, R.; Seto, M.; Yoda, Y.; Shimizu, K.; Machida, A.; Aoki, K. Erratum: Structural and Valence Changes of Europium Hydride Induced by Application of High-Pressure H2 (Physical Review Letters (2011) 107 (025501) DOI: 10.1103/PhysRevLett.107.025501). *Phys. Rev. Lett.* **2019**, *122* (17), 179901. https://doi.org/10.1103/PhysRevLett.122.179901.

(4) Semenok, D. V.; Zhou, D.; Kvashnin, A. G.; Huang, X.; Galasso, M.; Kruglov, I. A.; Ivanova, A. G.; Gavriliuk, A. G.; Chen, W.; Tkachenko, N. V.; Boldyrev, A. I.; Troyan, I.; Oganov, A. R.; Cui, T. Novel Strongly Correlated Europium Superhydrides. *J. Phys. Chem. Lett.* **2021**, *12* (1), 32–40. https://doi.org/10.1021/acs.jpclett.0c03331.

(5) Palasyuk, T.; Saxena, S.; Zaleski-Ejgierd, P.; Tkacz, M. High Pressure Studies of Terbium Trihydride. X-Ray, Raman and DFT Investigations. *J. Alloys Compd.* **2014**, *597*, 58–62. https://doi.org/10.1016/j.jallcom.2014.01.150.

(6) Meng, H.; Palasyuk, T.; Drozd, V.; Tkacz, M. Study of Phase Stability and Isotope Effect in Dysprosium Trihydride at High Pressure. *J. Alloys Compd.* **2017**, *722*, 946–952. https://doi.org/10.1016/j.jallcom.2017.06.181.

(7) Salke, N. P.; Davari Esfahani, M. M.; Yedukondalu, N.; Zhang, Y.; Kruglov, I. A.; Zhou, J.; Greenberg, E.; Prakapenka, V. B.; Liu, J.; Oganov, A. R.; Lin, J. F. Prediction and Synthesis of Dysprosium Hydride Phases at High Pressure. *Inorg. Chem.* **2020**, *59* (8), 5303–5312. https://doi.org/10.1021/acs.inorgchem.9b03078.

(8) Palasyuk, T.; Tkacz, M. Pressure-Induced Structural Phase Transition in Rare-Earth Trihydrides. Part I. (GdH3, HoH3, LuH3). *Solid State Commun.* **2005**, *133* (7), 481–486. https://doi.org/10.1016/j.ssc.2004.11.036.

(9) Kong, B.; Zhang, L.; Chen, X. R.; Zeng, T. X.; Cai, L. C. Structural Relative Stabilities and Pressure-Induced Phase Transitions for Lanthanide Trihydrides REH 3 (RE=Sm, Gd, Tb, Dy, Ho, Er, Tm, and Lu). *Phys. B Condens. Matter* **2012**, *407* (12), 2050–2057. https://doi.org/10.1016/j.physb.2012.02.003.

(10) Olsen, J. S.; Buras, B.; Gerward, L.; Johansson, B.; Lebech, B.; Skriver, H. L.; Steenstrup, S. A New High-Pressure Phase and the Equation of State of YbH2. *Phys. Scr.* **1984**, *29* (5), 503–507. https://doi.org/10.1088/0031-8949/29/5/016.
